# Atmospheric microplastic emissions from land and ocean

**DOI:** 10.1038/s41586-025-09998-6

**Published:** 2026-01-21

**Authors:** Ioanna Evangelou, Silvia Bucci, Andreas Stohl

**Affiliations:** Department of Meteorology and Geophysics, University of Vienna, Vienna, Austria

**Keywords:** Planetary science, Environmental sciences

## Abstract

Microplastics (MPs) are global pollutants^1^, yet their atmospheric distribution is poorly understood^2^. Although atmospheric MP measurements have become more abundant, estimates of emissions into the atmosphere vary by orders of magnitude^3,4^. Here we compile a global atmospheric MPs dataset and compare it with size-aligned MP model simulations. Our model simulations show two to four orders of magnitude overestimation of the measured global median atmospheric MP concentrations. Measured median concentrations over the ocean are 27 times lower than over the land (0.003 and 0.08 particles m^−3^, respectively). Applying a simple scaling method, we estimate that oceanic emissions are lower in number than land-based emissions. The total global land-based and oceanic emissions are 6.1 × 10^17^ (1.3 × 10^17^ to 1.1 × 10^18^) particles year^−1^ and 2.6 × 10^16^ (2.7 × 10^15^ to 5.0 × 10^16^) particles year^−1^, respectively. Our results indicate that fewer MP particles are emitted into the atmosphere than previously thought. Land sources dominate the number but not the mass emissions, indicating that MPs emission size distributions should be investigated further.

## Abstract (web summary)

A global atmospheric microplastics dataset is compiled, and comparison with size-aligned microplastic models in the literature indicate that fewer microplastic particles are emitted into the atmosphere than previously thought.

## Main

Plastic in the ocean was first reported in 1972 (ref. ^5^) and, since then, plastics have been found in all environmental compartments. MPs, defined as particles 1 μm to 5 mm in size, can be of primary origin (for example, tyre wear, abrasives and cosmetics) or formed by fragmentation of larger plastics. MPs have been detected in aquatic environments^6^, sediments^7^, sewage sludge^8^ and air^9^. Atmospheric emissions arise from tyre and brake wear, industrial and personal plastic use, resuspension from oceans^10^, soils^11^ and plastic-treated agricultural fields^12^. MPs can travel long distances in the atmosphere, reaching remote regions such as the Arctic and Antarctica^13,14^.

Nevertheless, measurements of atmospheric abundance and deposition are challenging because of the small particle sizes involved^15^. Reported atmospheric concentration levels are extremely variable. For instance, along the southeast coast of China, reports vary from 0.004 MP m^−3^ (ref. ^16^) to 190 MP m^−3^ (ref. ^17^). Atmospheric depositions of 50 MP m^−2^ day^−1^ were found in a megacity suburban region in China^18^, whereas 3,100 MP m^−2^ day^−1^ were reported at an urban location in the United Kingdom^19^. Measurement challenges include the lack of a universal sampling protocol, the unclear statement of minimum detectable sizes and size distributions and the incomplete characterization of particle shapes.

Quantifying the MP cycle requires accurate atmospheric emission estimates. Different emission datasets exist: bottom-up estimates based on human activity data and process modelling for resuspension, as well as top-down estimates constrained by atmospheric measurements. These estimates differ by orders of magnitude, even for global totals, and also report different relative source contributions. Brahney et al.^20^ and Evangeliou et al.^3^ combined in situ measurements, modelling and optimization to quantify the sources of atmospheric plastic. However, these studies used measurements from one specific region in the western United States to infer global emissions, a rather uncertain extrapolation. Both studies report that the ocean is the dominant source of MPs, with emissions of about 9 Tg year^−1^ for 0–100-μm (ref. ^20^) and 5–250-μm (ref. ^3^) size ranges, whereas a process-based study found only 0.1 Tg year^−1^ (10–280 μm) from the ocean^21^. Others reported even lower ocean emissions: 0.001 Tg year^−1^ for 1–60 μm (ref. ^10^) and 0.0007 Tg year^−1^ for 0.3–70 μm (ref. ^22^). Recently, model simulations using a global ocean emission of 0.0007 Tg year^−1^ reproduced specific MP observations well, suggesting that oceanic emissions of MPs are negligible compared with land-based sources^23^.

Here we compile existing atmospheric MP measurements from the literature (Methods section ‘Compilation of MP measurements’) to reveal whether they are compatible with estimated emission fluxes. We compare the measurements over ocean and over land and link them with the reported emissions. Furthermore, we establish quantitative source–receptor relationships between the emissions and the measured concentration and deposition values using a Lagrangian particle dispersion model (Methods section ‘Source–receptor modelling’). We reconcile the modelled and measured values and scale the emissions to improve the agreement.

## Emission estimates

**Extended Data Fig. 1:**
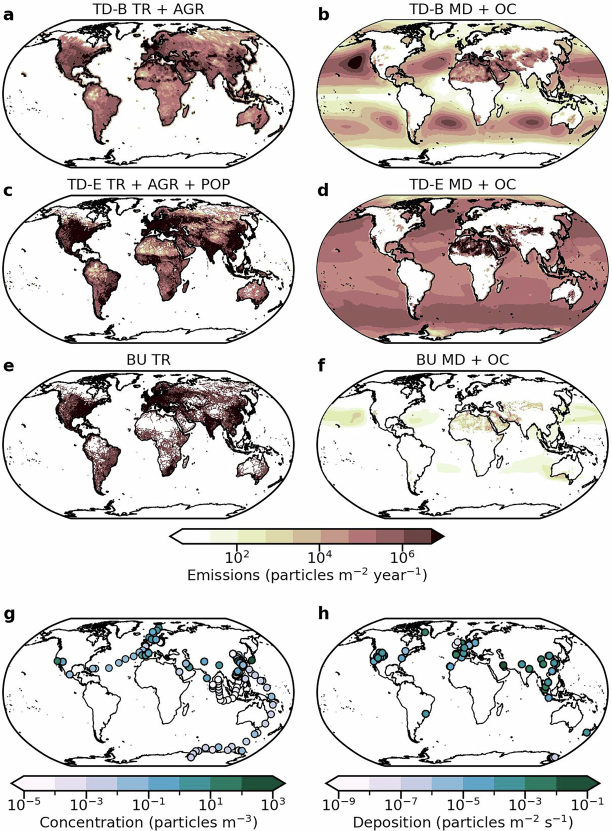
MP emissions from three inventories and measured MP concentrations and depositions. **a**, Sum of TD-B traffic (TR) and agricultural (AGR) MP emissions in particles m^−2^ year^−1^ for the size range 5–100 μm. **b**, Same as in **a** but for the sum of TD-B mineral dust (MD) and oceanic (OC) emissions. **c**, Same as in **a** but for the sum of TD-E TR, AGR and population (POP) emissions. **d**, Same as in **a** but for the sum of TD-E MD and OC emissions. **e**, Same as in **a** but for BU TR emissions. **f**, Same as in **a** but for the sum of BU MD and OC emissions. **g**, Compilation of measured MP concentrations (Supplementary Tables 2 and 3). **h**, Compilation of bulk, wet and dry deposition measurements (Supplementary Table 4).

We combined bottom-up estimates for three different emission sources^10,11,24^ into one bottom-up (BU) inventory and consider two published top-down (TD) emission estimates, referred to as TD-B^20^ and TD-E^3^, all three adjusted to the MP size range 5–100 μm (Methods section ‘Emission estimates’). The three emission cases, BU, TD-B and TD-E, report emissions that differ by orders of magnitude. For instance, the TD-B population emissions are zero, whereas those of TD-E are 3.0 (1.7–4.3) Tg year^−1^ (Supplementary Table 1). The TD-E oceanic emissions are 8.9 (7.8–9.9) Tg year^−1^, whereas the BU ocean emissions are only 0.001 (0.0003–0.005) Tg year^−1^. The BU bare arid soil emissions are 0.0001 (0.00004–0.00011) Tg year^−1^, whereas the corresponding TD-B estimate is 0.01 (0.005–0.06) Tg year^−1^. The three emission cases are depicted in Extended Data Fig. 1 as yearly particle number emission fluxes (Methods section ‘Emission estimates’). The total global land emissions are of similar magnitude for the three emission cases but their spatial patterns are different.

## Global MP measurements

We compiled 2,782 MP measurements of air concentration and deposition from 76 studies (Methods ‘Compilation of MP measurements’) and 283 different locations worldwide (Supplementary Fig. 2). The dataset includes 925 atmospheric concentration, 1,007 bulk deposition, 556 wet deposition and 294 dry deposition measurements collected during the period from 2014 to 2024 (Extended Data Fig. 1). The most commonly found MP shapes were fibres, fragments, microbeads, granules and films (Supplementary Tables 2–4). The analytical techniques used to confirm the chemical composition of the particles were mainly μ-Raman and μ-Fourier transform infrared spectroscopy. The reported sizes ranged from several microns to several mm.

## Atmospheric MP global analysis

**Fig. 1:**
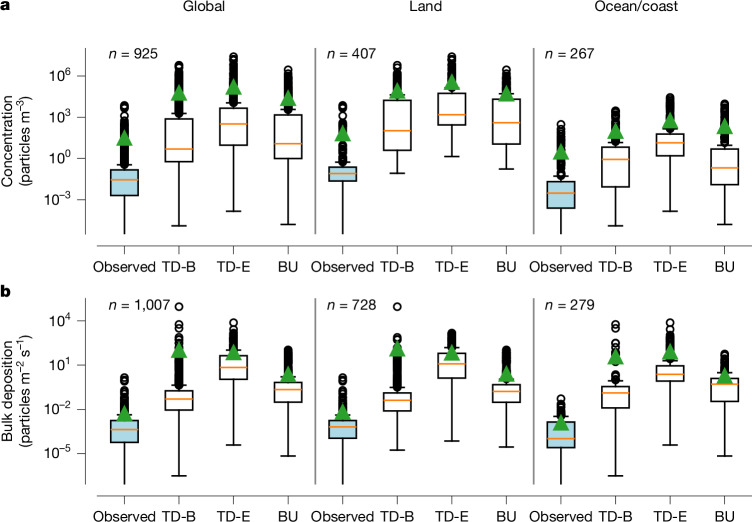
Frequency distributions of measured and simulated MP concentrations and depositions. **a**, Box and whisker plots representing the frequency distributions of measured (blue boxes) MP concentrations globally, over land excluding coastal regions and over the ocean, and the corresponding simulated values at the measurement locations (white boxes) for top-down (TD-B, TD-E) and bottom-up (BU) emission cases (Methods section ‘Emission estimates’). The boxes extend from the first quartile to the third quartile of the data, with a line at the median. The whiskers extend from the box by 1.5 times the interquartile range. The green triangles correspond to the mean values. *n* is the number of data points. **b**, Same as **a** but for MP bulk deposition.

The interquartile range (25th to 75th percentile) of measured MP atmospheric concentrations stretches from 0.002 to 0.1 particles m^−3^, with a median value of 0.03 particles m^−3^ and a mean value of 31 particles m^−3^ (Fig. 1 and Supplementary Tables 5 and 6). The bulk deposition ranges from 5.8 × 10^−5^ to 0.002 particles m^−2^ s^−1^, with a median of 0.0004 particles m^−2^ s^−1^ and a mean value of 0.005 particles m^−2^ s^−1^.

Excluding coastal areas, the median measured MP concentration over land is 0.08 particles m^−3^, whereas over the sea it is 0.003 particles m^−3^. Ignoring air mass transport as well as differences in vertical mixing, this suggests that the emission number flux densities over land are a factor of 27 higher than over the sea. Because the higher terrestrial emissions may also contribute substantially to the MP concentrations over the oceans, the real land/ocean emission density ratio may actually be substantially higher. For the TD-B, TD-E and BU cases, dividing the land and oceanic total fluxes (Supplementary Table 1) by the respective land and ocean surface areas, the corresponding mass (number) emission flux density ratios are 0.04, 0.9 and 835 (12, 48 and 148,450). Although this highlights the sensitivity of the results to the assumed size distributions and the conversion of mass to number values, it nevertheless seems likely that the TD inventories have a too low land/ocean emission ratio, whereas the opposite is true for the BU case.

The median observed MP bulk deposition over land is 0.0006 particles m^−2^ s^−1^. Over the sea, there exist only a few measurements near coastlines close to land sources with a median of 0.0001 particles m^−2^ s^−1^. Nevertheless, this confirms a dominance of land-based particle number emissions and indicates that most of the suspended MPs are deposited close to their terrestrial emission sources.

To compare modelled and measured MPs, it is necessary to align each modelled value to the measured size range reported for the corresponding observation (Supplementary Text 3). When comparing the global median modelled with the global median observed MP atmospheric concentrations and bulk deposition, we find that the model overestimates the observations by two to four orders of magnitude for all three emission cases (Fig. 1). Over land, the overestimation of atmospheric concentrations is particularly large. There the measured median is only 0.08 particles m^−3^, whereas simulated median values range from 105 particles m^−3^ for the TD-B case to 1,506 particles m^−3^ for the TD-E case (Supplementary Table 5). The deposition fluxes over land are not as strongly overestimated by the model as the concentrations; however, still overestimations of factors of 67–20,500 are found. Over the ocean, the measured median concentration of 0.003 particles m^−3^ is also strongly overestimated by the model (0.2 to 13.9 particles m^−3^ for the three emission cases) but not as strongly as over land.

For the global bulk deposition, the TD-B case (0.05 particles m^−2^ s^−1^) best reproduces the observed median value (0.0004 particles m^−2^ s^−1^); however, it still overestimates considerably. An interesting finding is that the BU case leads to similarly high overestimation over the ocean as the other two cases, even with an ocean emission flux that is almost four orders of magnitude lower. This implies that transport of overestimated land emissions causes the deposition overestimation over the sea.

Overall, there is little correlation between model-simulated and observed MP concentrations and depositions (Methods section ‘Statistics’ and Supplementary Fig. 3). The best correlation (*r* = 0.33) for the concentrations is found for the BU emission case (Supplementary Fig. 3c and Supplementary Table 7), which better reproduces the large observed land–sea contrasts than the other cases. Varying the modelled wet scavenging (Methods section ‘Source–receptor modelling’) does not change the simulated values substantially, indicating that MPs are primarily removed by settling-driven dry deposition.

## Scaled land and oceanic emissions

The poor spatial coverage and large variability of the available measurements, and the fact that the available emissions lead to strong overestimation of the measurements, do not provide a robust basis for improving the emissions with a formal inverse modelling approach or adjusting the emissions regionally. We therefore choose a simple scaling approach, with only two global scaling factors for land and ocean emissions (Methods section ‘Emission scaling’). As a basis, we use the BU emissions, as they are independent of the measurements and also produce the best correlation between measured and modelled concentrations.

**Fig. 2:**
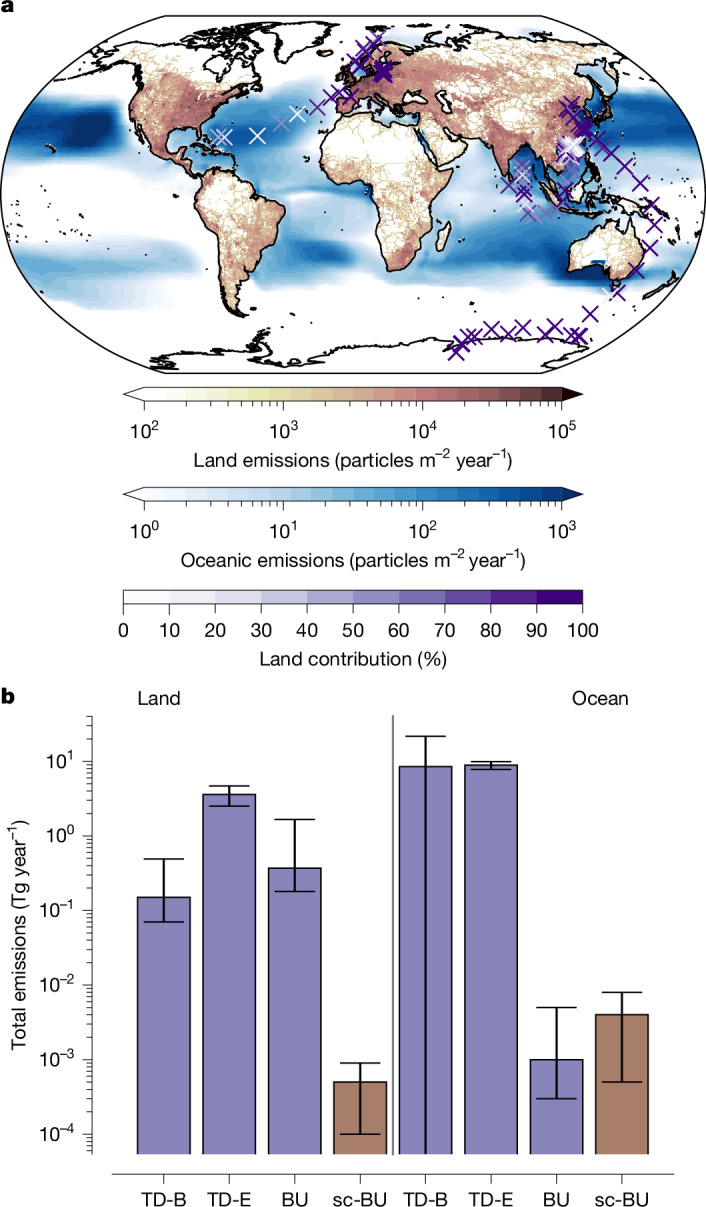
Scaled bottom-up emissions and comparison with top-down and bottom-up estimates. **a**, The scaled bottom-up number emissions (colour shading) and contribution of the land emissions to the simulated values over the ocean (coloured X markers) (Methods section ‘Emission scaling’). **b**, Total mass emissions with their 90% confidence intervals for land and ocean, for the TD-B, TD-E, BU and scaled BU (indicated as sc-BU) cases.

The resulting land and ocean scaling factors are 0.0013 (0.0003–0.0023) and 3.63 (0.37–6.88), respectively. The scaling leads to average terrestrial and oceanic emission fluxes of 2,660 (560–4,770) and 57 (6–107) particles m^−2^ year^−1^, respectively, with a significant difference between TD and BU and scaled BU terrestrial emissions (Fig. 2 and Supplementary Table 1). The total emitted MPs are 6.1 × 10^17^ (1.3 × 10^17^ to 1.1 × 10^18^) particles year^−1^ for land or 0.0005 (0.0001–0.0009) Tg year^−1^. For the ocean, the respective emissions are 2.6 × 10^16^ (2.7 × 10^15^ to 5.0 × 10^16^) particles year^−1^ or 0.004 (0.0005–0.008) Tg year^−1^.

Using the scaled BU emissions, the modelled values are in much better agreement with the measurements than when using any of the unscaled emissions (Supplementary Fig. 3 and Supplementary Tables 5, 6 and 7). The statistical performance measures for the deposition values also improved considerably, even though deposition data were not used for the scaling.

**Extended Data Fig. 2:**
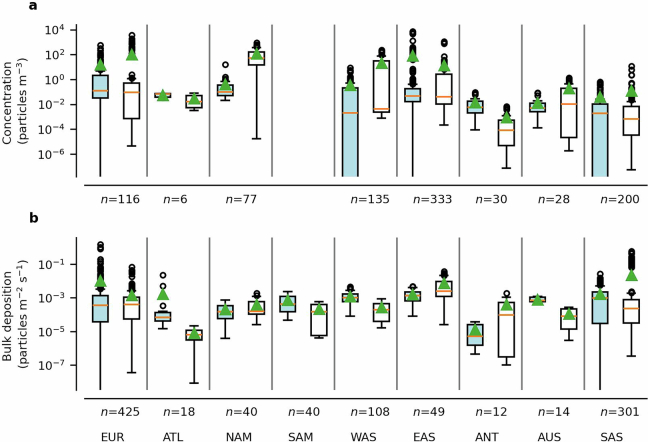
Frequency distributions of measured and simulated MP concentrations and depositions after the emission scaling. **a**, Box and whisker plots representing the frequency distributions of measured (blue boxes) and simulated (white boxes) MP concentrations using the scaled bottom-up emissions for the nine regions (Supplementary Fig. 2). The boxes extend from the first quartile to the third quartile of the data, with a line at the median. The whiskers extend from the box by 1.5 times the interquartile range. The green triangles correspond to the mean values. *n* is the number of data points. **b**, Same as **a** but for the MP bulk depositions.

Frequency distributions for the MP measured and scaled BU simulated values for nine regions (Supplementary Fig. 2) are shown in Extended Data Fig. 2 and for the unscaled TD-B and TD-E simulations in Supplementary Fig. 5. The highest median MP concentration (0.1 particles m^−3^) is observed over Europe, whereas the highest mean concentration (80 particles m^−3^) is recorded over East Asia. South Asia exhibits the lowest measured median MP concentration. In terms of bulk deposition, East Asia shows the highest measured median value (0.001 particles m^−2^ s^−1^), whereas Antarctica has the lowest observed value (5 × 10^−6^ particles m^−2^ s^−1^). Overall, the model using scaled BU emissions reproduces the regional variability, although there are exceptions (Supplementary Figs. 6–14). For instance, over North America, deposition values are relatively well reproduced but atmospheric concentrations are strongly overestimated. Terrestrial emissions are the prevalent source in all regions except for Antarctica, in which oceanic emissions dominate.

## Discussion and conclusions

Aligning the modelled values to the measured size ranges shows that the emissions are overestimated by both top-down and bottom-up inventories, mainly because of overestimated terrestrial emissions. After the emission scaling, we estimate global MP emissions of 0.0045 (0.0006–0.0089) Tg year^−1^ in the size range 5–100 μm (Supplementary Table 1). Fu et al.^4^ calculated global MP emissions of 0.32 Tg year^−1^ for sizes of 0.3–70 μm, which is still two orders of magnitude larger than our estimate.

The estimated MP land emissions differ significantly from those of previous TD and BU studies (Fig. 2b and Supplementary Table 1). Fu et al.^4^ reported 0.15 Tg year^−1^ emitted from terrestrial sources, compared with our scaled emissions of 0.0005 Tg year^−1^. We further estimate annual global MP marine emissions of 0.004 (0.0005–0.008) Tg for particles of 5–100 μm, which is relatively close to our BU estimate but much lower than the TD-E one (Fig. 2b and Supplementary Table 1). Studies upscaling experimental results have reported 2 × 10^−6^ (<10 μm), 8 × 10^−4^ (0.3–70 μm) and 0.1 (10–280 μm) Tg year^−1^ (refs. ^21,22,25^), revealing a systematic dependence on the size range considered, as also discussed by Shaw et al.^21^. Shaw et al.’s rather high emission estimate includes particles of sizes that may not remain airborne long enough to be transported in the atmosphere over substantial distances. Overall, when considering differences in size ranges, our scaled emissions are broadly consistent with the above process-based studies. On the other hand, previous TD studies have reported much higher marine emissions^3,4,20^ that are not consistent with the available MP measurement data over the open ocean. This overestimate can probably be explained by inversion artefacts produced when using terrestrial MP data to constrain oceanic sources.

Simulations using our scaled emissions still show systematic deviations from the measurements (Supplementary Fig. 3d,h). With more observation data becoming available in the future, this could be improved by conducting regional or emission-sector-specific emission scaling. Eventually, inverse modelling could help constrain the emissions. However, with the available measurement data at present and highly uncertain emissions, this approach is not considered robust enough. Progress in estimating emissions is also limited by the poorly known emission factors for various human activities and the lack of information on emission size distributions. This can make the conversion of mass values to number values, and vice versa, uncertain by several orders of magnitude and makes comparisons with measurements difficult. Future research should focus on resolving the MP size distribution of both emissions and measured atmospheric MPs and on extending the measured size range to smaller MPs and nanoplastics.

## Methods

### Emission estimates

So far, no atmospheric emission inventory exists that covers all known MP sources. Traffic emissions are considered one of the largest primary MP environmental sources^26,27^. We used the bottom-up emissions as described in ref. ^24^ (Supplementary Text 1), which compiles emissions from tyre wear, brake wear, road markings and road surface wear. Recently, Bucci et al.^10^ and Evangelou et al.^11^ estimated the resuspension emissions from the ocean and from bare arid soils, respectively. Both studies reported bottom-up estimates based on process modelling of the respective source. We combined the traffic, ocean and bare arid soil emissions and refer to this emission case as ‘bottom-up’ (BU). Notice that potentially important emissions are missing from this inventory (for example, emissions from clothing, industrial sources and so on). No reliable bottom-up emission estimates exist for these sources but we assume that their spatial distribution is similar to the traffic emissions. Traffic emissions may thus serve as a proxy for all primary MP emissions.

In the study of Brahney et al.^20^, emissions from the ocean, traffic, mineral dust, agricultural dust and population activities were estimated on the basis of proxies, such as population density, sea spray and mineral dust emissions. An atmospheric transport model was then fed with these emissions and the resulting simulated values were compared with in situ measurements of MP deposition in the western United States. The emissions were then optimized top-down. Following the study of Brahney et al., Evangeliou et al.^3^ presented a different top-down emission estimate using the same measurements but a different modelling and inversion approach. We refer to these two top-down (TD) emission inventories as TD-B and TD-E for Brahney et al. and Evangeliou et al., respectively.

All three emission datasets were regridded to a 0.5° × 0.5° spatial resolution. The TD-B and TD-E emission estimates are available at yearly and 6-h time resolution, respectively. BU traffic-related MP emissions are given at yearly and the oceanic and resuspension MP emissions at 6-h resolution.

The total mass emissions of each sector were weighted to the four size bins 5–10, 10–25, 25–50 and 50–100 μm used for atmospheric transport modelling (Supplementary Table 1), on the basis of one size distribution per sector (Supplementary Text 1 and Supplementary Fig. 1). Thus, the emission magnitude of the different emission cases can be compared, as the size distribution influence is the same for every estimate.

MP emissions are mostly reported in mass units, whereas most atmospheric MP measurements are reported as number concentrations, which requires a conversion that is sensitive to the underlying assumed size distributions. The emissions were converted from particle mass to particle number units using the geometric mean size of each size bin and assuming a spherical shape, except for the TD-E population sector, for which fibres were assumed.

### Compilation of MP measurements

Measurements of atmospheric MP concentration and bulk, wet and dry deposition were collected from reports in the literature. Studies were included only if they reported the sampling location and dates, the identified particle sizes and shapes and the measured air concentration or deposition of MPs, expressed as number (or mass) per volume of air or, for deposition measurements, per m^2^ per sampling period or day, respectively.

The MP measurement dataset is available with information about the study (author name, year of publication, DOI), the location (longitude, latitude and, if applicable, height above sea level), time of sampling (start and end date), type of measurement (air concentration, bulk, wet or dry deposition) and measured value (in number and mass units). The shapes and sizes of the particles are also included, as well as the code of the region in which we classified the measurement (Supplementary Fig. 2) and the type of sampling region (land, coastal, sea).

Because most studies reported the abundance of MPs in number of particles found, we converted all of the mass data values into number values (Supplementary Text 2). The resulting MP values for concentration and deposition in units of particles m^−3^ and particles m^−2^ s^−1^ are shown in Extended Data Fig. 1.

### Source–receptor modelling

To establish quantitative relationships between the emission sources and the measured air concentrations and deposition values, we used the Lagrangian particle dispersion model FLEXPART version 11 (ref. ^28^). FLEXPART offers an efficient option to calculate emission sensitivities through backward-in-time calculations from the location and times of air concentration^29,30^ and deposition measurements^31^. The emission sensitivity matrices provide quantitative relationships between potential sources and measurements. When multiplied by the spatially resolved emission fluxes from the three inventories, modelled air concentrations and deposition values that correspond in space and time with the measured values can be calculated.

The model was driven with ERA5 meteorological reanalysis data^32^ with hourly time resolution and 0.5° × 0.5° spatial resolution with 138 vertical levels. FLEXPART accounts for in-cloud and below-cloud wet scavenging, dry deposition, as well as shape-dependent and size-dependent gravitational settling. For simulating the in-cloud scavenging, the cloud condensation nuclei and ice nuclei efficiencies were set to 0.05 and 0.15, respectively, as a base case^33^. Cloud condensation nuclei and ice nuclei efficiencies of 0.001 and 0.01 as well as 0.5 and 0.8 (minimum and maximum scavenging case) were also used for calculating the sensitivity of the simulation results to the wet scavenging.

For air concentration measurements, we released 100,000 virtual particles within a layer of 0–50 m above ground level at the location and time of each measurement sample (for shipboard measurements, we released at a height of 30 m). For deposition values, we traced wet and dry deposition separately backward by releasing 100,000 virtual particles within the lowest 30 m for dry deposition and 1,000,000 particles within the whole atmospheric column for wet deposition^31^. For total deposition, the emission sensitivity fields for dry and wet deposition were combined. We tracked the particles backward in time for at least 60 days, which corresponds to several times the expected atmospheric lifetime of micrometre-sized particles.

We simulated four particle size bins, 5–10, 10–25, 25–50 and 50–100 μm. For the simulations, we specified the shape according to the dominant shape reported by the respective experimental study or, if the percentage contribution of several shapes was reported, we weighted the measured values with the reported percentages and simulated the different shapes.

Fibres and fragments were simulated as cylinders with an aspect ratio (that is, length-to-diameter ratio) corresponding to the measurements or with an aspect ratio of 40 and 2, respectively, if no information was available. Films were simulated as square-shaped particles with a thickness (smallest dimension) of 5 μm (ref. ^34^). All other morphologies were assumed to be spherical. The particle density was set to 1,200 kg m^−3^. The simulated values were aligned to correspond with the measured size range of the respective study, using the simulated size distribution (Supplementary Text 3).

### Statistics

To determine the agreement between observed and modelled values, we used the following statistical measures: the Pearson correlation coefficient (*r*), the fractional bias (FB), the factor of exceedance (FOEX), the root mean squared error (RMSE) and the fraction of predictions within a factor of ten of the observations (FAC10).

For *N* pairs of observations (${C}_{{{\rm{o}}}_{i}}$) and simulations (${C}_{{{\rm{s}}}_{i}}$), the statistical measures are defined as:

1 $$r=\frac{{\sum }_{i=1}^{N}({C}_{{{\rm{s}}}_{i}}-\overline{{C}_{{\rm{s}}}})({C}_{{{\rm{o}}}_{i}}-\overline{{C}_{{\rm{o}}}})}{\sqrt{{\sum }_{i=1}^{N}{({C}_{{{\rm{s}}}_{i}}-\overline{{C}_{{\rm{s}}}})}^{2}{\sum }_{i=1}^{N}{({C}_{{{\rm{o}}}_{i}}-\overline{{C}_{{\rm{o}}}})}^{2}}}$$

2 $${\rm{FB}}=\frac{2}{N}\mathop{\sum }\limits_{i=1}^{N}\frac{({C}_{{{\rm{s}}}_{i}}-{C}_{{{\rm{o}}}_{i}})}{({C}_{{{\rm{s}}}_{i}}+{C}_{{{\rm{o}}}_{i}})}$$

3 $${\rm{FOEX}}\,( \% )=\left[\frac{N({C}_{{{\rm{s}}}_{i}} > {C}_{{{\rm{o}}}_{i}})}{N}-0.5\right]\times 100$$

4 $${\rm{RMSE}}=\sqrt{\frac{{\sum }_{i=1}^{N}{({C}_{{{\rm{s}}}_{i}}-{C}_{{{\rm{o}}}_{i}})}^{2}}{N}}$$

FAC10 is defined as the fraction of data for which:

5 $$0.1\le \frac{{C}_{{{\rm{s}}}_{i}}}{{C}_{{{\rm{o}}}_{i}}}\le 10.0$$

in which $\overline{{C}_{{\rm{o}}}}$ is the mean of the observed values, $\overline{{C}_{{\rm{s}}}}$ is the mean of the simulated values and *N*(${C}_{{{\rm{s}}}_{i}}$ > ${C}_{{{\rm{o}}}_{i}}$) is the number of overpredictions. It is true that *r* ∈ [−1, 1], FB ∈ [−2, 2], FOEX ∈ [−50%, 50%] and RMSE ∈ [0, *∞*].

The Pearson correlation coefficient *r* measures the linear correlation between the observed and simulated values. The FB evaluates the mean relative difference between predicted and observed values and is bounded between −2 and 2, with 0 indicating no bias. FOEX shows whether a model tends to overpredict or underpredict the measurements. A FOEX value equal to −50% means that all of the values are underpredicted, whereas a value of 50% shows that all of the values are overpredicted. RMSE is a variation metric, and a value of 0 would indicate a perfect fit to the data. FAC10 gives the fraction of simulated values that are within a factor of 10 of the observed values.

### Emission scaling

Because observed median MP concentrations over the ocean are only 4% (3%–6%) of those over land, measurements over land are unlikely to be strongly influenced by the advection of oceanic MPs. Therefore, we first scale the terrestrial emissions such that the mean modelled and observed concentrations over land are identical and subsequently scale the oceanic emissions in the same way but using only observations that are not heavily influenced by the land emissions.

We determined the ratio *R* of observed and BU-model-simulated mean atmospheric concentrations over land (*n* = 407; Supplementary Fig. 4):

6 $$R=\frac{\overline{\,{{\rm{o}}{\rm{b}}{\rm{s}}}_{{\rm{l}}{\rm{a}}{\rm{n}}{\rm{d}}}}}{\overline{\,{{\rm{s}}{\rm{i}}{\rm{m}}}_{{\rm{l}}{\rm{a}}{\rm{n}}{\rm{d}}}}}$$

in which $\overline{{{\rm{obs}}}_{{\rm{land}}}}$ and $\overline{{{\rm{sim}}}_{{\rm{land}}}}$ are the mean observed and simulated values, respectively. Subsequently, we scaled the sum of the terrestrial BU emissions with *R*, while preserving the BU emission spatial distribution. This way, we avoid artificially reducing uncertainties in regions lacking observations. A 90% two-sided confidence interval was derived for the emission scaling factor based on error propagation of the underlying uncertainties of the ratio (Supplementary Text 4).

Using the scaled terrestrial emissions, we simulated again the concentrations over the ocean using BU emissions and identified the measurement samples in which the oceanic MP contribution was larger than the scaled terrestrial contribution (that is, oceanic contribution larger than 50%). This left 33 measurements over the open ocean (Supplementary Fig. 4). We then subtracted the modelled terrestrial contribution from the measurements and scaled the oceanic BU emissions with the ratio of the mean measurement residual and the modelled oceanic contribution. The uncertainty of the oceanic scaling factor was calculated in the same way as for land. Notice that the relative uncertainty of the scaling factor is larger for the ocean than for land, owing to the smaller sample size and sparser spatial coverage. The difference in our scaled emissions compared with previous studies^3,20^ originates primarily from our much larger and globally more representative measurement dataset, including many measurements from East Asia and over the ocean. In fact, the mean modelled oceanic emission contribution to the measurement locations used in Brahney et al.^20^ and Evangeliou et al.^3^ is minor (3% and 0.25% for TD-B and TD-E emission cases, respectively). This indicates that these measurements made in the continental interior are not sufficient to constrain the oceanic MP source and that MP measurements over the open sea are necessary. Further reasons for the difference in our scaled emissions are methodological differences and our alignment of measured and modelled size distributions.

## Online content

Any methods, additional references, Nature Portfolio reporting summaries, source data, extended data, supplementary information, acknowledgements, peer review information; details of author contributions and competing interests; and statements of data and code availability are available at 10.1038/s41586-025-09998-6.

## Supplementary information

Supplementary information This file contains Supplementary Methods, Supplementary Tables and Supplementary Figs. Supplementary Methods includes Supplementary Texts 1–4; estimation of traffic emissions and selection of emission size distributions; particle mass to number conversion method; simulated values alignment; and emission scaling factor uncertainty. Supplementary Tables includes Supplementary Tables 1–7; number and mass emissions for the different emission cases; information on measurement studies; simulated and measured median and mean values; and statistical metrics. Supplementary Figs. includes Supplementary Figs. 1–14; emission number size distributions; measurement locations and domains used for the regional analysis; scatter plots and box plots of simulated and measured values; measurements used for the emission scaling; and regional maps comparing BU with scaled BU cases.

Source Data Figure 1

Source Data Extended Data Figure 2

## Data Availability

The MP compiled dataset is available in the Zenodo repository^35^ (10.5281/zenodo.17660114). The top-down TD-B, TD-E, bottom-up BU and scaled BU MP emissions are also available in the same repository.

## Code availability

The Lagrangian particle dispersion model FLEXPART version 11 is open access at https://www.flexpart.eu/. The FLEXPART configuration files needed to reproduce the simulations of this study can be found in the Zenodo repository^35^ (10.5281/zenodo.17660114).

## References

[1] Hale, R. C., Seeley, M. E., La Guardia, M. J., Mai, L. & Zeng, E. Y. A global perspective on microplastics. *J. Geophys. Res. Oceans* **125**, e2018JC014719 (2020).

[2] Beaurepaire, M., Dris, R., Gasperi, J. & Tassin, B. Microplastics in the atmospheric compartment: a comprehensive review on methods, results on their occurrence and determining factors. *Curr. Opin. Food Sci.* **41**, 159–168 (2021).

[3] Evangeliou, N., Tichý, O., Eckhardt, S., Zwaaftink, C. G. & Brahney, J. Sources and fate of atmospheric microplastics revealed from inverse and dispersion modelling: from global emissions to deposition. *J. Hazard. Mater.* **432**, 128585 (2022). 35299104 10.1016/j.jhazmat.2022.128585

[4] Fu, Y. et al. Modeling atmospheric microplastic cycle by GEOS-Chem: an optimized estimation by a global dataset suggests likely 50 times lower ocean emissions. *One Earth* **6**, 705–714 (2023).

[5] Carpenter, E. J. & Smith, K. L. Plastics on the Sargasso Sea surface. *Science* **175**, 1240–1241 (1972). 5061243 10.1126/science.175.4027.1240

[6] Isobe, A. et al. A multilevel dataset of microplastic abundance in the world’s upper ocean and the Laurentian Great Lakes. *Microplastics Nanoplastics* **1**, 16 (2021).

[7] Büks, F. & Kaupenjohann, M. Global concentrations of microplastics in soils – a review. *SOIL* **6**, 649–662 (2020).

[8] Rolsky, C., Kelkar, V., Driver, E. & Halden, R. U. Municipal sewage sludge as a source of microplastics in the environment. *Curr. Opin. Environ. Sci. Health* **14**, 16–22 (2020).

[9] Goßmann, I. et al. Occurrence and backtracking of microplastic mass loads including tire wear particles in northern Atlantic air. *Nat. Commun.* **14**, 3707 (2023). 37349297 10.1038/s41467-023-39340-5 PMC10287736

[10] Bucci, S., Richon, C. & Bakels, L. Exploring the transport path of oceanic microplastics in the atmosphere. *Environ. Sci. Technol.* **58**, 14338–14347 (2024). 39078311 10.1021/acs.est.4c03216 PMC11325545

[11] Evangelou, I., Tatsii, D., Bucci, S. & Stohl, A. Atmospheric resuspension of microplastics from bare soil regions. *Environ. Sci. Technol.* **58**, 9741–9749 (2024). 38767840 10.1021/acs.est.4c01252 PMC11155246

[12] Mbachu, O., Jenkins, G., Pratt, C. & Kaparaju, P. A new contaminant superhighway? A review of sources, measurement techniques and fate of atmospheric microplastics. *Water Air Soil Pollut.* **231**, 85 (2020).

[13] Evangeliou, N. et al. Atmospheric transport is a major pathway of microplastics to remote regions. *Nat. Commun.* **11**, 3381 (2020). 32665541 10.1038/s41467-020-17201-9 PMC7360784

[14] Aves, A. R. et al. First evidence of microplastics in Antarctic snow. *Cryosphere* **16**, 2127–2145 (2022).

[15] Peries, S. D., Sewwandi, M., Sandanayake, S., Kwon, H.-H. & Vithanage, M. Airborne transboundary microplastics–a swirl around the globe. *Environ. Pollut.* **353**, 124080 (2024). 38692389 10.1016/j.envpol.2024.124080

[16] Wang, X. et al. Efficient transport of atmospheric microplastics onto the continent via the East Asian summer monsoon. *J. Hazard. Mater.* **414**, 125477 (2021). 33647626 10.1016/j.jhazmat.2021.125477

[17] Liao, Z. et al. Airborne microplastics in indoor and outdoor environments of a coastal city in Eastern China. *J. Hazard. Mater.* **417**, 126007 (2021). 33992007 10.1016/j.jhazmat.2021.126007

[18] Huang, Y. et al. Atmospheric transport and deposition of microplastics in a subtropical urban environment. *J. Hazard. Mater.* **416**, 126168 (2021). 34492944 10.1016/j.jhazmat.2021.126168

[19] Jenner, L. C. et al. Outdoor atmospheric microplastics within the Humber Region (United Kingdom): quantification and chemical characterisation of deposited particles present. *Atmosphere* **13**, 265 (2022).

[20] Brahney, J. et al. Constraining the atmospheric limb of the plastic cycle. *Proc. Natl Acad. Sci. USA* **118**, e2020719118 (2021). 33846251 10.1073/pnas.2020719118 PMC8072239

[21] Shaw, D. B., Li, Q., Nunes, J. K. & Deike, L. Ocean emission of microplastic. *PNAS Nexus* **2**, pgad296 (2023). 37795272 10.1093/pnasnexus/pgad296 PMC10547021

[22] Yang, S. et al. Constraining microplastic particle emission flux from the ocean. *Environ. Sci. Technol. Lett.* **9**, 513–519 (2022).

[23] Yang, S., Brasseur, G., Walters, S., Lichtig, P. & Li, C. W. Y. Global atmospheric distribution of microplastics with evidence of low oceanic emissions. *npj Clim. Atmos. Sci.* **8**, 81 (2025).

[24] Tatsii, D., Gasparini, B., Evangelou, I., Bucci, S. & Stohl, A. Do microplastics contribute to the total number concentration of ice nucleating particles? *J. Geophys. Res. Atmos.* **130**, e2024JD042827 (2025).

[25] Harb, C., Pokhrel, N. & Foroutan, H. Quantification of the emission of atmospheric microplastics and nanoplastics via sea spray. *Environ. Sci. Technol. Lett.* **10**, 513–519 (2023).

[26] Sommer, F. et al. Tire abrasion as a major source of microplastics in the environment. *Aerosol Air Qual. Res.* **18**, 2014–2028 (2018).

[27] Hann, S. et al. Investigating options for reducing releases in the aquatic environment of microplastics emitted by (but not intentionally added in) products. Technical report, Eunomia Research & Consulting Ltd. https://eunomia.eco/reports/ (2018).

[28] Bakels, L. et al. FLEXPART version 11: improved accuracy, efficiency, and flexibility. *Geosci. Model Dev.* **17**, 7595–7627 (2024).

[29] Seibert, P. & Frank, A. Source-receptor matrix calculation with a Lagrangian particle dispersion model in backward mode. *Atmos. Chem. Phys.* **4**, 51–63 (2004).

[30] Stohl, A., Forster, C., Frank, A., Seibert, P. & Wotawa, G. Technical note: the Lagrangian particle dispersion model FLEXPART version 6.2. *Atmos. Chem. Phys.* **5**, 2461–2474 (2005).

[31] Eckhardt, S. et al. Source–receptor matrix calculation for deposited mass with the Lagrangian particle dispersion model FLEXPART v10.2 in backward mode. *Geosci. Model Dev.* **10**, 4605–4618 (2017).

[32] Hersbach, H. et al. The ERA5 global reanalysis. *Q. J. R. Meteorol. Soc.* **146**, 1999–2049 (2020).

[33] Grythe, H. et al. A new aerosol wet removal scheme for the Lagrangian particle model FLEXPART v10. *Geosci. Model Dev.* **10**, 1447–1466 (2017).

[34] Chen, Q. et al. Aging simulation of thin-film plastics in different environments to examine the formation of microplastic. *Water Res.* **202**, 117462 (2021). 34343870 10.1016/j.watres.2021.117462

[35] Evangelou, I., Bucci, S. & Stohl, A. Atmospheric microplastic emissions from land and ocean. *Zenodo*10.5281/zenodo.17660114 (2025). 10.1038/s41586-025-09998-6 PMC12851922 41565806

