# Supplementary material for: Atmospheric microplastic emissions from land and ocean
Source: Nature. 2026 Jan 21;649(8099):1186–9. doi: 10.1038/s41586-025-09998-6 (PMC12851922; doi:10.1038/s41586-025-09998-6)
Supplement: Supplementary file 1 — This file contains Supplementary Methods, Supplementary Tables and Supplementary Figs. Supplementary Methods includes Supplementary Texts 1–4; estimation of traffic emissions and selection of emission size distributions; particle mass to number conversion method; simulated values alignment; and emission scaling factor uncertainty. Supplementary Tables includes Supplementary Tables 1–7; number and mass emissions for the different emission cases; information on measurement studies; simulated and measured median and mean values; and statistical metrics. Supplementary Figs. includes Supplementary Figs. 1–14; emission number size distributions; measurement locations and domains used for the regional analysis; scatter plots and box plots of simulated and measured values; measurements used for the emission scaling; and regional maps comparing BU with scaled BU cases. [file 41586_2025_9998_MOESM1_ESM.pdf]

nature portfolio

https://doi.org/10.1038/s41586-025-09998-6

Supplementary information

Atmospheric microplastic emissions from land and ocean

In the format provided by the
authors and unedited

Atmospheric microplastic emissions from land and ocean

Ioanna Evangelou[1*], Silvia Bucci[1] and Andreas Stohl[1]

[1*]Department of Meteorology and Geophysics, University of Vienna, Josef-Holaubek-Platz 2, Vienna, 1090, Vienna, Austria.

*Corresponding author(s). E-mail(s): ioanna.evangelou@univie.ac.at;
Contributing authors: silvia.bucci@univie.ac.at;
andreas.stohl@univie.ac.at;

1 Supplementary Methods

1.1 Supplementary Text 1

Estimation of traffic-related bottom-up BU emissions and size distributions of emission sectors. The traffic-related BU emissions include microplastic (MP) emissions from tire wear, brake wear, road markings, and polymer-modified bitumen (PMB) road wear. Emission factors were collected from literature to calculate the annual emissions per capita, and the resulting total global emissions were disaggregated based on the paved road length (GRIP4 global road database [35]). We assume that 0.81 kg of tire wear per person per year is emitted based on Kole et al., 2017 [36]. This means that 6350 kt tire emissions are occurring globally, and assuming that 50% of it is plastic, this corresponds to 3175 kt MP emitted into the environment per year. Brake wear is estimated to be 175 kt per year based on the study of Evangeliou et al. [37]. For road markings, the value of 63 grams MP per person per year (median of studies for different countries: Norway, Sweden, Denmark, Finland, Iceland, Europe, OSPAR countries [Belgium, Denmark, Finland, France, Germany, Iceland, Ireland, Luxembourg, Netherlands, Norway, Portugal, Spain, Sweden, Switzerland, UK], Austria, Germany, Latin America, Croatia, Poland) [38, 39] was used. This leads to 494 kt of MP emitted per year globally. Regarding asphalt, it is reported that 73,950 kt of bitumen per year is used in roads around the globe [40]. Around 15% of it is PMB, thus, 11,092.5 kt per year of PMB is used. Assuming that the PMB contains 5 wt% plastic, then 555 kt of plastic is contained in this type of asphalt. Assuming that the plastic is abraded 100%, we estimate that 555 kt of MP are emitted per year from PMB asphalt. Thus, the total traffic emissions into the environment are estimated to be 4400 kt annually. We assume that 10% of these emissions can be suspended in the atmosphere, which results in annual global MP emissions into the atmosphere of 440 kt or 0.44 Tg.

One unique emission weighting size distribution is selected for each emission source sector, which is thought to represent the respective emission process best. The total mass emissions of the sector are weighted with the selected mass size distribution. After weighting, the mass emissions of each size bin are converted to number emissions. The resulting normalized number size distribution is shown in Supplementary Fig. 1.

Regarding the MP resuspension from bare arid soils, the size distribution of Evangelou et al. [41] (0-70 μm) is used. It is based on the mineral dust size distribution from the literature, as it is plausible that the MP resuspension size distribution is rather similar.

MPs emitted from agricultural soil surfaces are expected to have the same size distribution as the MPs in arid regions. Thus, for the agricultural sector, Evangelou et al.'s size distribution (0-70 μm) is used.

For the resuspension from oceans, the emission size distribution of Bucci et al. [42] is used. The emission estimation in the aforementioned study is based on process modeling of the resuspension, taking into account the MP measurements and a measurement-based MP size distribution at the ocean surface, and a sea salt emission scheme.

Regarding the population source, Evangeliou et al. [43] is the only study that reports MP emissions from population activities, which we also use. The size bins in their study correspond to the length of fibers, which we convert to volume-equivalent particle diameters based on their reported fiber length and diameter.

For the traffic-related particles, it is reported that the dominant modal sizes are less than 100 μm [44–47]. Therefore, the size distribution of Brahney et al. [48] covering 0-100 μm is selected in this case.

1.2 Supplementary Text 2

Particle mass to number conversion for measurements. To calculate the number concentrations and depositions for those studies that reported mass-based values, we first calculate the mass of one particle based on the available information. For the particle density (ρ) we choose the value of 1200 kg m^{-3}, a common value for MPs [49]. The shape selected for the conversion is the most abundant shape reported in the study or, when the percentages of different shapes are stated, all shapes are taken into consideration. If particulate matter (PM) is reported, then the selected shape is a sphere.

Regarding the particle size, we use the average reported size wherever it is available. In any other case, the geometric mean is used for the reported size range (e.g., $[s_1, s_2]$, $s = \sqrt{s_1 s_2}$). For fibers and fragments, if not mentioned, the particle diameter D is calculated from the reported size (i.e., the longest dimension) assuming that the particle has an aspect ratio AR (longest dimension to diameter ratio) of 40 and 2, respectively. For spheres, the reported size is considered as the particle diameter D. Films are treated as thin squares characterized by their three dimensions L (long), I (intermediate), and S (small). The L dimension is set to the reported size. The S dimension is set to 5 μm [50]. Lastly, the I dimension is calculated to be $L/\sqrt{2}$, corresponding to the side of a square with a diagonal equal to L.

The volume of one particle is calculated based on its dimensions and then multiplied by ρ to calculate its mass m.

$$m_{sphere} = \rho \frac{4}{3} \pi (\frac{D}{2})^3$$

$$m_{fiber/fragment} = \rho \pi (\frac{D}{2})^2 (AR) D$$

$$m_{film} = \rho \pi (\frac{S}{2})^2 L$$

Finally, to calculate the respective number concentration or deposition, the measured mass equivalent is divided by the mass m of one particle.

1.3 Supplementary Text 3

Alignment of simulated values. One simulated value sim_i is calculated for each one of the 5-10, 10-25, 25-50, and 50-100 µm particle size bins with bin widths Δx_i. We first fit a power law $y = aD^b$, where D is the particle size, to the simulated values. Then the integral of the fitted distribution for the measured equivalent diameter size range $[d_{eq1}, d_{eq2}]$ is calculated and divided by the respective integral for the 5-100 µm range. To align from the 5-100 µm range to the measured size range, the resulting fraction is multiplied by the total simulated value:

$$sim_{aligned} = \frac{\int_{d_{eq1}}^{d_{eq2}} aD^b \, dD}{\int_5^{100} aD^b \, dD} \sum_i sim_i \Delta x_i$$

1.4 Supplementary Text 4

Uncertainty calculation of emission scaling factor. The standard error of the ratio $R = \frac{\bar{x}}{\bar{y}}$ using the error propagation method, assuming independence between the numerator and the denominator, is:

$$\mathrm{SE}(R) \approx \sqrt{\left(\frac{\mathrm{SE}(\bar{x})}{\bar{y}}\right)^2 + \left(\frac{\bar{x}\mathrm{SE}(\bar{y})}{\bar{y}^2}\right)^2}$$

where $\bar{x}$ and $\bar{y}$ are the means of numerator and denominator, respectively, and $\mathrm{SE}(\bar{x})$ and $\mathrm{SE}(\bar{y})$ are their standard errors.

The 90% two-sided confidence interval of R is then:

$$(R - 1.64\mathrm{SE}(R), R + 1.64\mathrm{SE}(R))$$

2 Supplementary Tables

Supplementary Table 1: Total global mass and particle number MP emissions per sector for the 5-100 µm size range, for the emission estimates top-down TD-B, TD-E, bottom-up BU and scaled BU. The emission uncertainty ranges (90% confidence intervals) are given in the parentheses.

	TD-B_m	TD-E_m	BU_m ($Tg\ yr^{-1}$)	scaled BU_m	TD-B_n	TD-E_n ($\times 10^{15}$ particles yr^{-1})	BU_n	scaled BU_n
Ocean	8.5 (0-21.7)	8.9 (7.8-9.9)	0.001 (0.0003-0.005)	0.004 (0.0005-0.008)	51,000 (0-130,000)	53,000 (47,000-59,000)	7.3 (2.2-36)	26 (2.7-50)
Population	0	3.0 (1.7-4.3)	-		0	26,000 (15,000-37,000)	-	
Traffic	0.09 (0.06-0.10)	0.26 (0.23-0.29)	0.37 (0.18-1.65)		110,000 (73,000-120,000)	300,000 (260,000-340,000)	480,000 (230,000-2,100,000)	
Agriculture	0.05 (0-0.33)	0.25 (0.22-0.28)	-		130,000 (0-860,000)	590,000 (510,000-670,000)	-	
Mineral dust	0.01 (0.005-0.060)	0.08 (0.07-0.09)	0.0001 (0.00004-0.00011)		34,000 (17,000-200,000)	200,000 (170,000-230,000)	190 (76-210)	
Total land	0.15 (0.07-0.49)	3.59 (2.51-4.67)	0.37 (0.18-1.66)	0.0005 (0.0001-0.0009)	270,000 (120,000-880,000)	1,120,000 (770,000-1,400,000)	480,000 (230,000-2,200,000)	610 (130-1,100)

Supplementary Table 2: Atmospheric MP concentration measurements from ship cruises

	Study	Sample Year	Region	Size (μm)	Shape	Method	Units
1	Trainic et al., 2020 [51]	2016	N. Atlantic	>5	elongated	μ-Raman	number
2	Liu et al., 2019 [52]	2018	W. Pacific	20-2000	fiber,fragment	μ-FTIR	mass
3	Allen et al., 2020 [53]	2018	France Atlantic coast	5-38	-	μ-Raman	number
4	Liu et al., 2020 [54]	2018	Pacific	16-2986	fiber	μ-FTIR	number
5	Ferrero et al., 2022 [55]	2019	Baltic	1-2500	fiber,fragment	μ-FTIR	number
6	Ding et al., 2021 [56]	2019	China	50-2210	fiber,fragment,granule	μ-FTIR	number
7	Chen et al., 2023 [57]	2019-2020	China to Antarctica	>20	fiber,fragment	μ-FTIR	mass
8	Li et al., 2022 [58]	2020	China	87-2566	fiber,fragment	μ-FTIR	number
9	Wang et al., 2021 [59]	2020	China	155-949	fiber,fragment,film	μ-FTIR	number
10	Wang et al., 2020 [60]	2020	India-China	59-2251	fiber,fragment	μ-FTIR	number
11	Gossmann et al., 2023 [61]	2021	N. Atlantic	>5	-	py-GC/MS	mass
12	Gossmann et al., 2023 [62]	2021	Sweden	>3	-	py-GC/MS	mass

FTIR: Fourier-Transform Infrared spectroscopy

py-GC/MS: pyrolysis-Gas Chromatography/Mass Spectrometry

Supplementary Table 3: Atmospheric MP concentration measurements

	Study	Sample Year	Region	Size (µm)	Shape	Method	Units
1	Martynova et al., 2024 [63]	2015-2017	Saudi Arabia	183-11877	fiber	Raman/SRS	number
2	Allen et al., 2021 [64]	2017	France	3.5-60	fiber,fragment	µ-Raman	number
3	Akhabarizadeh et al., 2021 [65]	2017	Iran	8-1000	fiber,fragment,film	µ-Raman	number
4	Abbasi et al., 2019 [66]	2017	Iran	10-100	fiber,fragment	SEM/EDS	number
5	Li et al., 2020 [67]	2018	China	5-200	fiber	SEM-EDX	number
6	Gonzalez-Pleiter et al., 2021 [68]	2018-2019	Spain	42-1709	fiber,fragment	µ-FTIR	number
7	Gaston et al., 2020 [69]	2019	California	25-2061	fiber,fragment	µ-Raman/ µ-FTIR	number
8	Prata et al., 2020 [70]	2019	Portugal	26-1928	fiber	stereomicroscopy	number
9	Liu et al., 2019 [71]	2019	China	12-2191	fiber,fragment,bead	µ-FTIR	number
10	Abbasi et al., 2023 [72]	2019	Iran	10-1000	fiber	SEM-EDX/ µ-Raman	number
11	Zhu et al., 2021 [73]	2019	China	>5	fiber,fragment	µ-FTIR	number
12	Shruti et al., 2022 [74]	2020	Mexico	>40	fiber,fragment	ATR-FTIR/ SEM-EDX	number
13	Rao et al., 2024 [75]	2020-2021	China	>20	fiber,fragment	FTIR	number
14	Chandrakanthan et al., 2023 [76]	2020-2021	Arizona	>5	fiber	µ-Raman	number
15	Kyriakoudes and Turner, 2023 [77]	2021	England	>30	fiber, fragment	FTIR	number
16	Yuan et al., 2023 [78]	2021	China	30-50	fiber	µ-FTIR	number
17	Xu et al., 2024 [79]	2021	China	8-1542	fiber,fragment,film	SEM-EDX/ FTIR/ Raman	number
18	Romarate et al., 2023 [80]	2021	Philippines	159-4807	fiber,fragment,film	FTIR	number
19	Sharaf et al., 2024 [81]	2022	Pakistan	10-500	fiber,fragment	ATR-FTIR/ µ-Raman/ SEM-EDX	number
20	Liu et al., 2025 [82]	2022	China	20-271	fragment	LDIR	number
21	Morioka et al., 2024 [83]	2022	Japan	>4.7	-	py-GC/MS	mass
22	Yuan et al., 2023 [84]	2021	China	13-334	fiber,fragment	µ-FTIR	number
23	Kaushik et al., 2024 [85]	2016,2020	India	20-5000	fiber,film,fragment	µ-FTIR	number
24	Perera et al., 2022 [86]	2021	Sri Lanka	67-4919	fiber,fragment	µ-FTIR	number
25	Liao et al., 2021 [87]	2019	China	5-100	fragment,fiber	µ-FTIR	number
26	Guo et al., 2024 [88]	2023	China	20-200	fragment,fiber,pellet	LDIR	number
27	Long et al., 2024 [89]	2023	China	50-2000	fiber,fragment	µ-FTIR	number
28	Nafea et al., 2025 [90]	2023-2024	China	20-4985	fiber,fragment	FTIR	number

SRS: Stimulated Raman Scattering spectroscopy

SEM: Scanning Electron Microscopy

EDX: Energy Dispersive X-ray spectroscopy

FTIR: Fourier-Transform Infrared spectroscopy

ATR: Attenuated Total Reflectance spectroscopy

py-GC/MS: pyrolysis-Gas Chromatography/Mass Spectrometry

LDIR: Laser Direct Infrared spectroscopy

Supplementary Table 4: Atmospheric MP deposition measurements

	Study	Sample Year	Region	Size (µm)	Shape	Method	Units
1	Dris et al., 2015 [91]	2014	France	1000-5000	fiber	microscopy	number
2	Dris et al., 2016 [92]	2014-2015	France	50-600	fiber	µ-FTIR	number
3	Cai et al., 2017 [93]	2016	China	200-1200	fiber	µ-FTIR	number
4	Klein and Fischer 2019 [94]	2017-2018	Germany	>5	fiber,fragment	µ-Raman	number
5	Allen et al., 2019 [95]	2017-2018	France	1-300	fiber,fragment,film	µ-Raman	number
6	Roblin et al., 2020 [96]	2017-2018	Ireland	>50	fiber	µ-Raman	number
7	Szewc et al., 2021 [97]	2017-2018	Poland	>5	fiber,fragment,film	ATR FTIR	number
8	Allen et al., 2022 [98]	2017-2018	France	10-400	fiber,fragment,film	µ-Raman	mass
9	Brahney et al., 2020 [99]	2017-2019	USA	4-3000	fiber,particles	FTIR	number
10	Wright et al., 2020 [100]	2018	UK	20-2500	fiber,non-fiber	µ-FTIR	number
11	Kernchen et al., 2022 [101]	2018	Germany	11-1945	fiber,fragment	Raman/ µ-FTIR	number
12	Hamilton et al., 2021 [102]	2018	Arctic	200-2000	fiber,fragment	Raman/ FTIR	number
13	Huang et al., 2021 [103]	2018-2019	China	1-2000	fiber,fragment,film,bead	µ-FTIR	number
14	Thinh et al., 2020 [104]	2018-2019	Vietnam	>100	fiber,fragment	microscopy	number
15	Truong et al., 2021 [105]	2018-2019	Vietnam	301-4872	fiber,fragment	FTIR-ATR	number
16	Purwiyanto et al., 2022 [106]	2018-2019	Indonesia	358-925	fiber,fragment,foam	FTIR	number
17	Abbasi and Turner, 2021 [107]	2019-2020	Iran	>20	fiber	µ-Raman	number
18	Welsh et al., 2022 [108]	2019-2020	Canada	20-4980	fiber,fragment	µ-Raman	number
19	Amato-Lourenço et al., 2022 [109]	2019-2020	Brasil	50-2725	fiber,fragment, film,granule	FTIR-ATR	number
20	Jenner et al., 2022 [110]	2019-2020	UK	11-30	fiber,fragment,film	µ-FTIR	number
21	Kernchen et al., 2024 [111]	2019-2020	Germany	11-130	fiber, fragment	Raman/ µ-FTIR	number
22	Liu et al., 2022 [112]	2020	China	>50	fiber,fragment	FTIR	number
23	Rao et al., 2024 [75]	2020-2021	China	>20	fiber,fragment	FTIR	number
24	Hee et al., 2023 [113]	2020-2021	Malaysia	5-50	fiber,fragment	µ-FTIR	number
25	Abbasi, 2021 [114]	2021	Iran	>20	fiber	SEM-EDX/ µ-Raman	number
26	Jia et al., 2022 [115]	2021	Shanghai	32-4960	fiber,film,granule	µ-FTIR	number
27	Kyriakoudes and Turner, 2023 [77]	2021	England	>30	fiber	FTIR	number
28	Zhang et al., 2023 [116]	2021-2022	Beijing	30-5543	fiber,fragment,film,bead	µ-FTIR	number
29	Edo et al., 2023 [117]	2021-2022	Spain	44-1720	fiber,fragment,film	µ-FTIR	number
30	Lu et al., 2024 [118]	2021-2022	Beijing	10-1000	fiber	µ-FTIR	number
31	Parashar and Hait, 2023 [119]	2022	India	1-4624	fiber,fragment	FESEM- -EDX/ FTIR	number
32	Aves et al., 2024 [120]	2020	New Zealand	50-3550	fiber,fragment	µ-FTIR	number
33	Beaurepaire et al., 2024 [121]	2020-2021	France	25-100	-	µ-FTIR	number
34	Ankit et al., 2024 [122]	2021-2022	India	67-2320	film,fragment,fiber	Raman	number
35	Chen et al., 2024 [123]	2021	China	20-4165	fiber,non-fiber	µ-FTIR	number
36	Elnahas et al., 2025 [124]	2023	United States	39-3743	fiber,fragment,film	Raman	number
37	Illuminati et al., 2024 [125]	2020	Antarctica	5-400	fragment,fiber	Raman	number
38	Logvina et al., 2024 [126]	2022-2024	Portugal	12-25	non-fiber,fiber	microscopy	number

FTIR: Fourier-Transform Infrared spectroscopy

ATR: Attenuated Total Reflectance spectroscopy

SEM: Scanning Electron Microscopy

EDX: Energy Dispersive X-ray spectroscopy

FESEM: Field Emission Scanning Electron Microscopy

Supplementary Table 5: Median global values for observed and simulated MP concentrations (in particles m^{-3}) and bulk depositions (in particles m^{-2} s^{-1}) for the three emission estimates and the scaled emissions. The values are for the base scavenging case.

	measured	TD-B	TD-E	BU	scaled BU
Concentration					
global	0.03	4.9	326.2	11.8	0.02
land	0.08	105.4	1,506.4	401.2	0.6
ocean	0.003	0.8	13.9	0.2	0.0005
Bulk deposition					
global	0.0004	0.05	7.0	0.2	0.0003
land	0.0006	0.04	12.3	0.2	0.0002
coast	0.0001	0.1	2.4	0.5	0.0007

Supplementary Table 6: Mean global values for observed and simulated MP concentrations (in particles m^{-3}) and bulk depositions (in particles m^{-2} s^{-1}) for the three emission estimates and the scaled emissions. The values are for the base scavenging case.

	measured	TD-B	TD-E	BU	scaled BU
Concentration					
global	31	59,280	160,730	24,220	31
land	65	82,350	357,540	51,470	65
ocean	3	100	570	220	0.3
Bulk deposition					
global	0.005	105.2	73.1	2.4	0.008
land	0.007	130.8	69.3	2.6	0.005
coast	0.001	38.3	82.9	1.9	0.02

Supplementary Table 7: Statistical metrics (Methods 2.4) for agreement of simulated and observed values for the three emissions estimates and the scaled emissions. The values are for the base scavenging case.

	TD-B	TD-E	BU	scaled BU
Concentration (n = 925)				
r	0.09	0.09	0.33	0.34
FB	1.6	1.9	1.7	0.4
FOEX (%)	41.7	48.2	43.7	9.0
RMSE (particles m^{-3})	4.1×10^5	1.4×10^6	1.4×10^5	3.7×10^2
FAC10 (%)	12.1	5.8	11.7	35.2
Deposition (n = 1857)				
r	-0.001	-0.003	-0.003	-0.003
FB	1.9	2.0	1.9	0.3
FOEX (%)	48.9	49.3	49.3	8.8
RMSE (particles m^{-2} s^{-1})	1.4×10^4	5.9×10^3	5.3×10^2	2.2
FAC10 (%)	8.5	0.9	2.7	53.3

3 Supplementary Figures

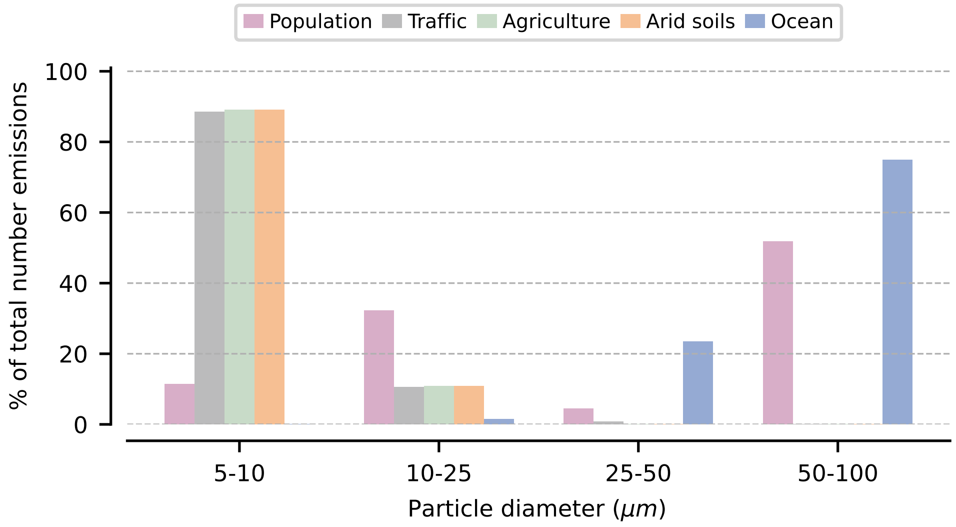

Supplementary Figure 1: **Number emission size distributions.** Normalized size distributions used for each emission source of the top-down TD-B, TD-E and bottom-up BU emission cases.

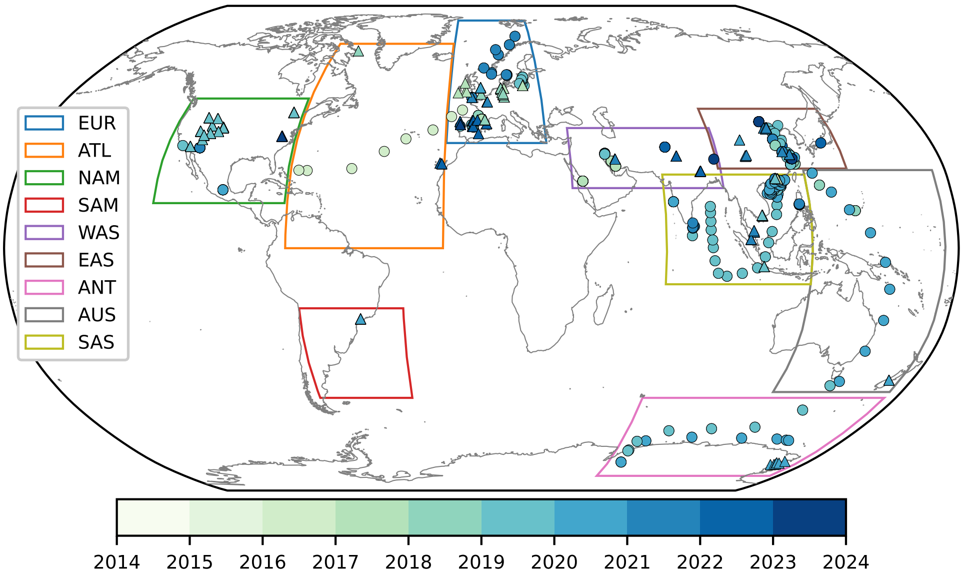

Supplementary Figure 2: MP atmospheric concentration and deposition measurement locations around the globe. The marker color corresponds to the year of sampling. The circles show concentration measurements and the triangles deposition data. The nine rectangles correspond to the different regions used for the analysis.

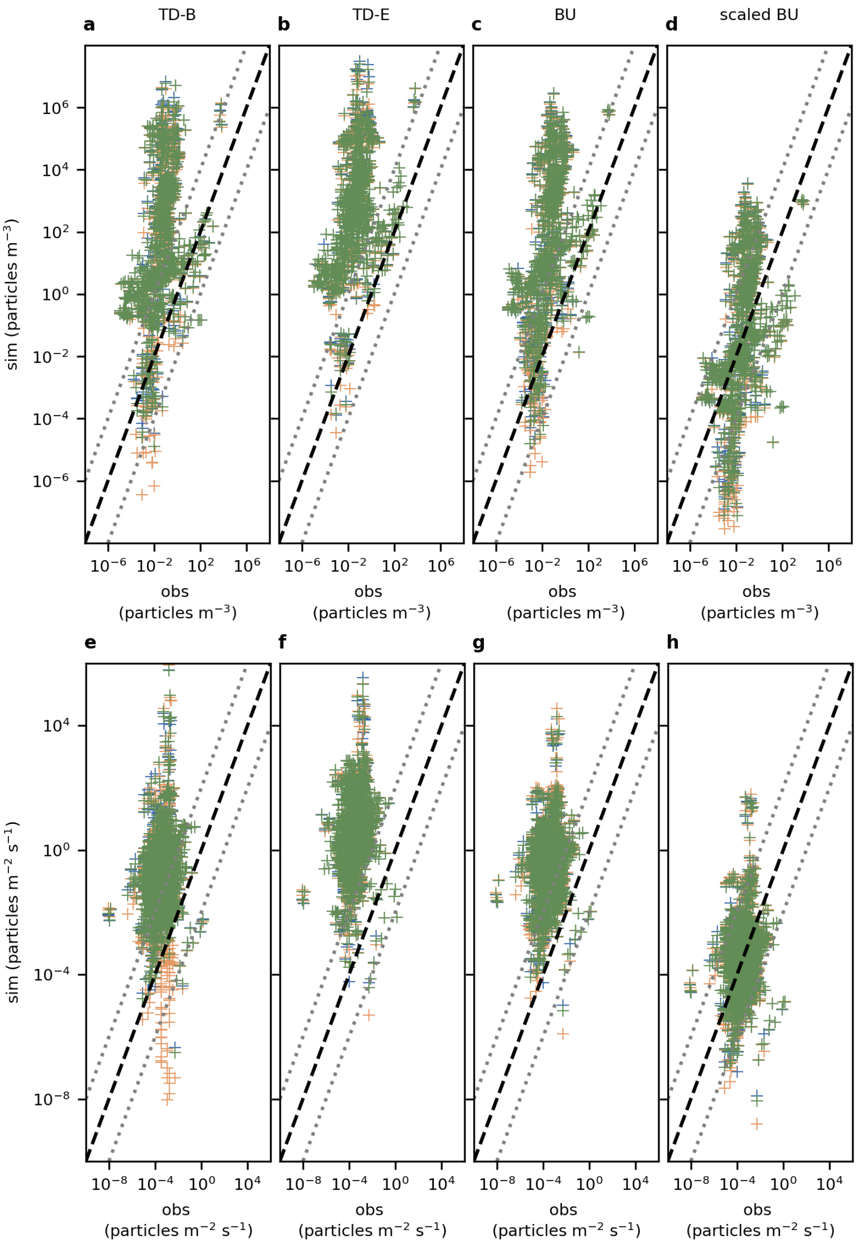

Supplementary Figure 3: **Scatter plots of observed (obs) and simulated (sim) atmospheric concentrations and bulk, wet and dry deposition values globally for the three emission cases (TD-B, TD-E, BU) and the scaled emissions (scaled BU). a-d**, MP concentrations for the TD-B, TD-E, BU and scaled BU cases and **e-h**, MP deposition for the TD-B, TD-E, BU and scaled BU cases. The green, blue, and orange crosses correspond to the base, minimum, and maximum scavenging cases, respectively. The bold dashed line corresponds to the 1:1 line, while the dotted lines correspond to the 100:1 and 1:100 lines.

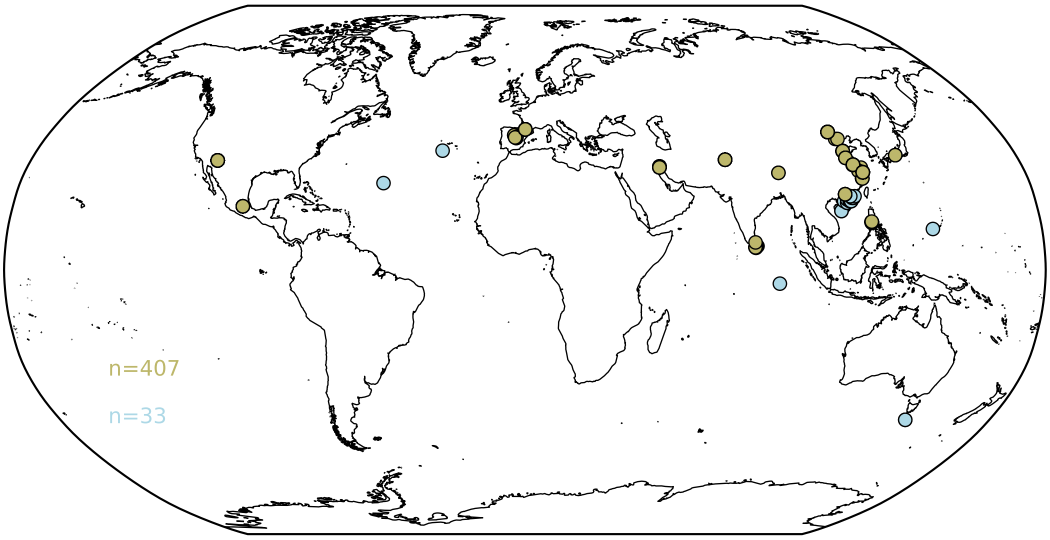

Supplementary Figure 4: **Emission scaling measurements.** Measurements used for the scaling of the land emissions (brown circles) and the oceanic emissions (blue circles) of the bottom-up BU case.

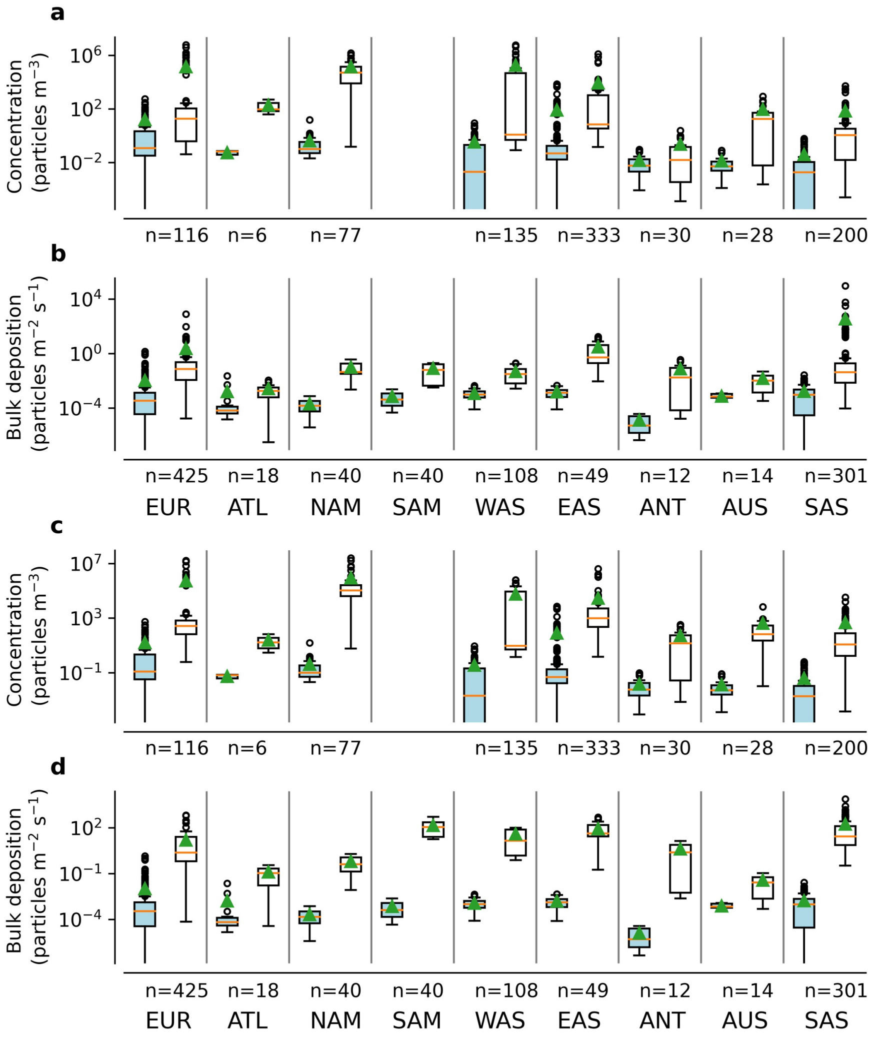

Supplementary Figure 5: **Frequency distributions of measured and simulated microplastic concentrations and depositions.** **a**, Box-and-whiskers plots representing the frequency distributions of measured (blue boxes) MP concentrations and the corresponding simulated values at the measurement locations (white boxes) for the top-down TD-B emissions for the nine regions. The green triangles represent the mean values. n is the number of data points. The boxes extend from the first quartile (Q1) to the third quartile (Q3) of the data, with a line at the median. The whiskers extend from the box by 1.5 times the interquartile range (IQR). **b**, Same as **a** but for MP depositions for the TD-B emissions. **c**, Same as **a** but for MP concentrations for the TD-E emissions. **d**, Same as **a** but for MP depositions for the TD-E emissions.

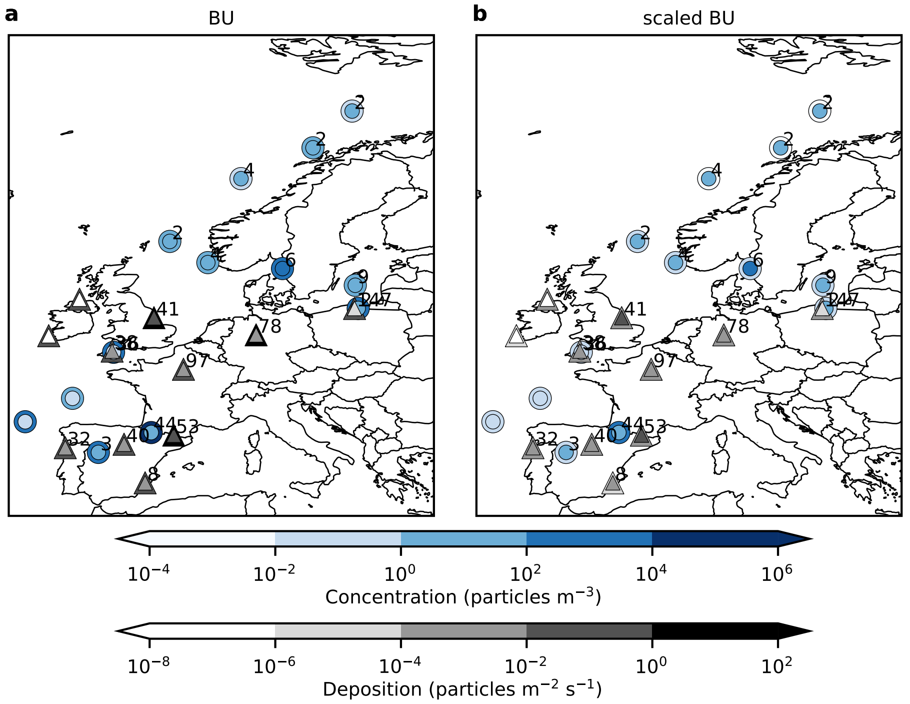

Supplementary Figure 6: **Comparison of measured and simulated values for the region of Europe (EUR). a**, Comparison for the bottom-up BU. Concentrations are represented with circles and deposition with triangles. In each symbol, the inner color filling corresponds to the measured value, while the outer color filling shows the simulated value. The symbols correspond to data averaged over 5°×5° regions. The number to the top right of each symbol indicates the number of points averaged. **b**, Same as in **a**, but for the scaled BU emissions.

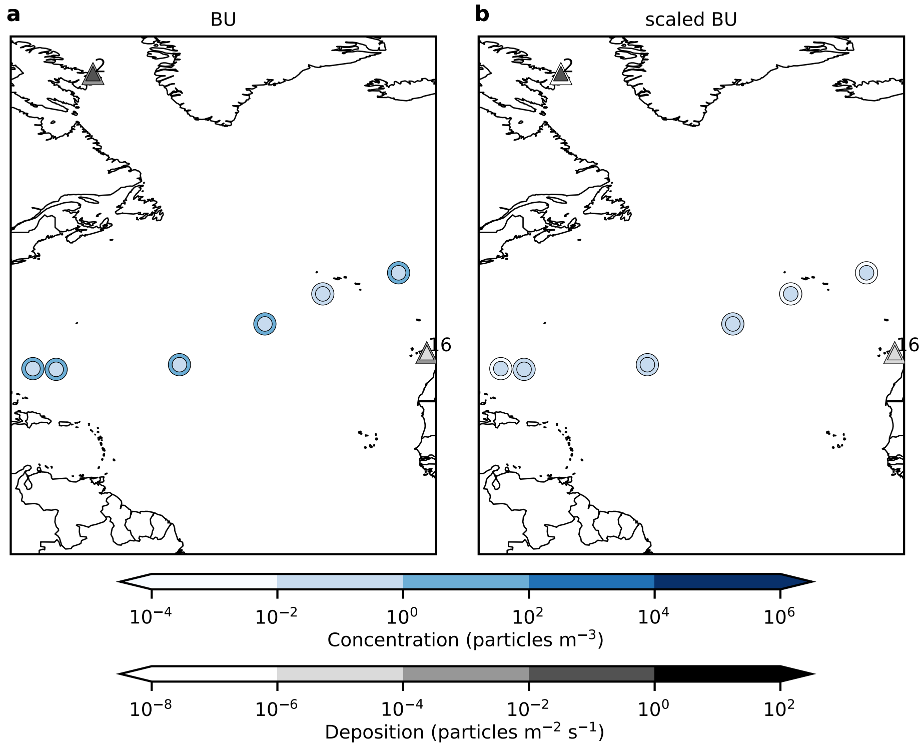

Supplementary Figure 7: Same as Supplementary Figure 6, but for the region of Atlantic (ATL).

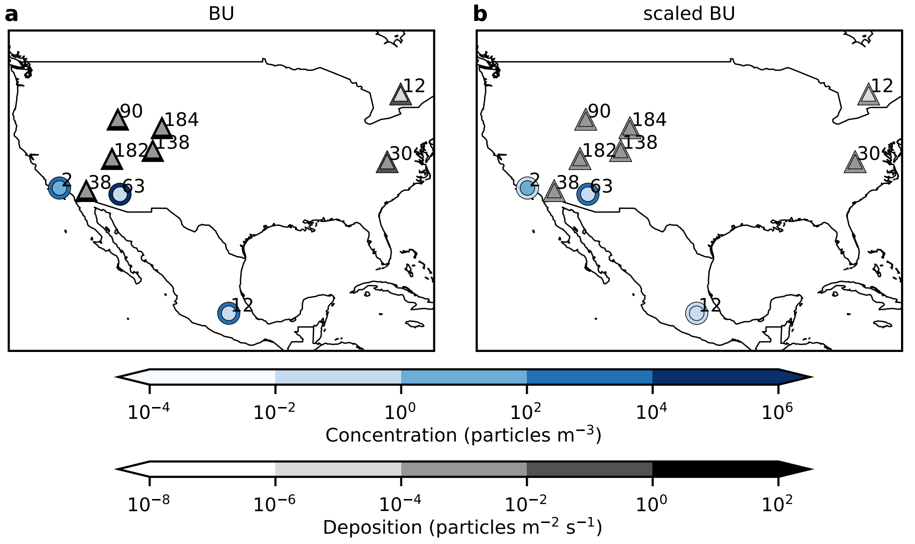

Supplementary Figure 8: Same as Supplementary Figure 6, but for the region of North America (NAM).

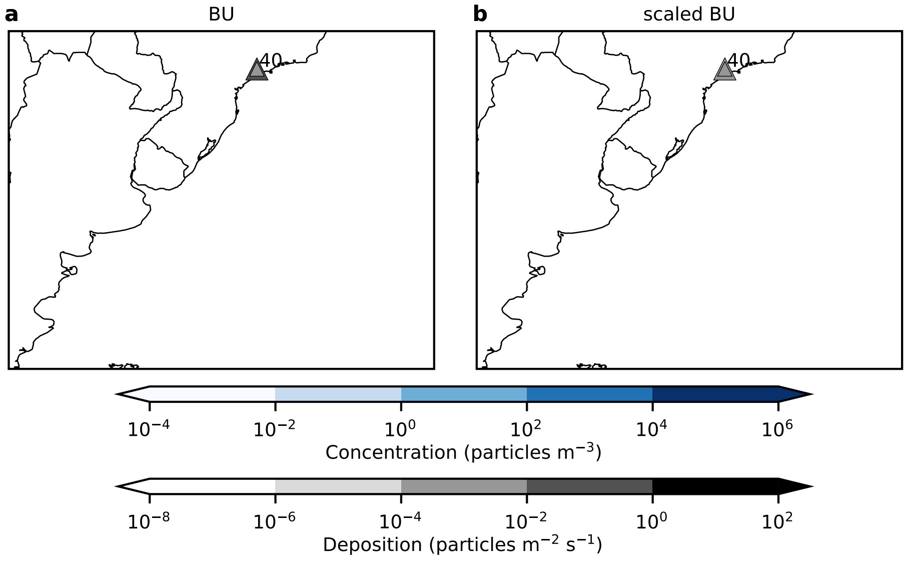

Supplementary Figure 9: Same as Supplementary Figure 6, but for the region of South America (SAM).

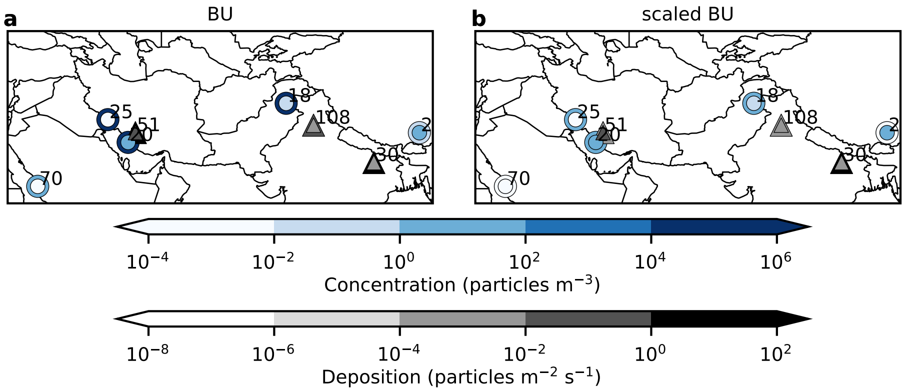

Supplementary Figure 10: Same as Supplementary Figure 6, but for the region of West Asia (WAS).

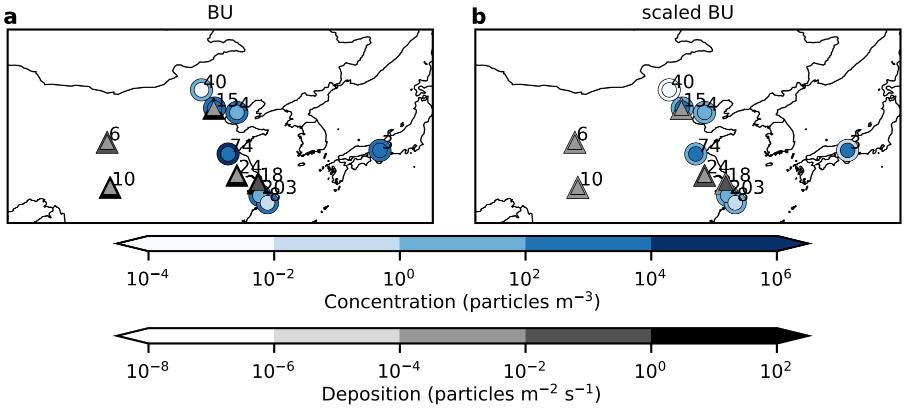

Supplementary Figure 11: Same as Supplementary Figure 6, but for the region of East Asia (EAS).

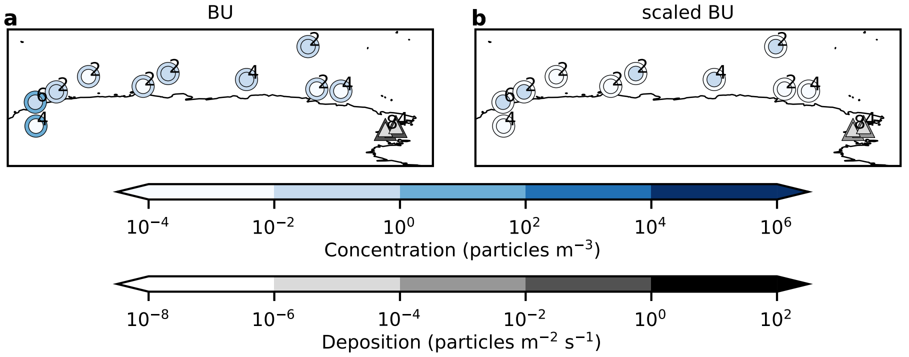

Supplementary Figure 12: Same as Supplementary Figure 6, but for the region of Antarctica (ANT).

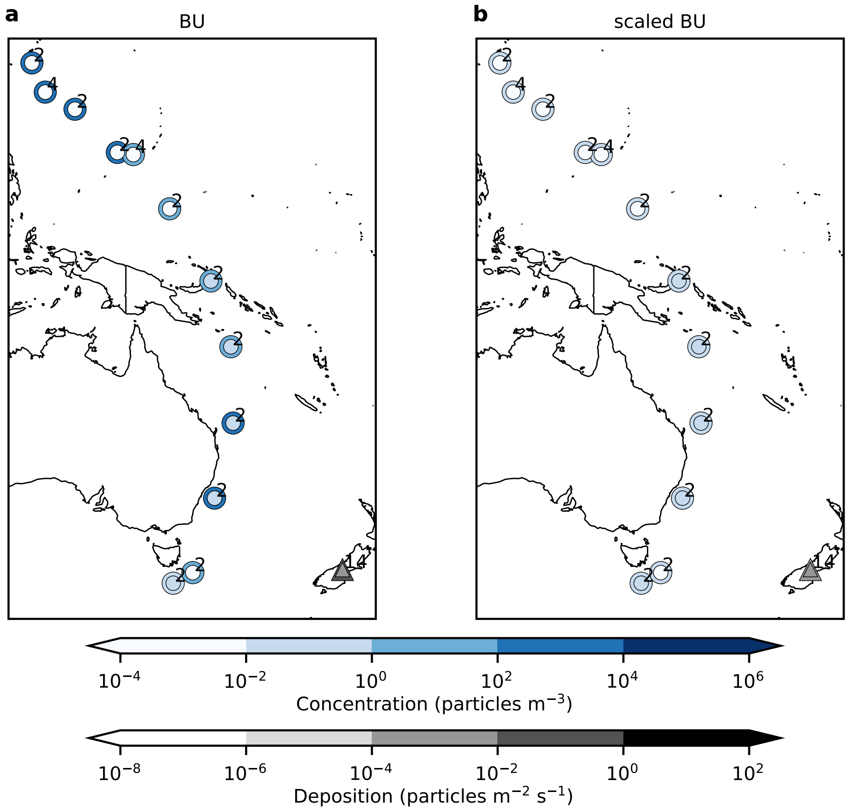

Supplementary Figure 13: Same as Supplementary Figure 6, but for the region of Australia (AUS).

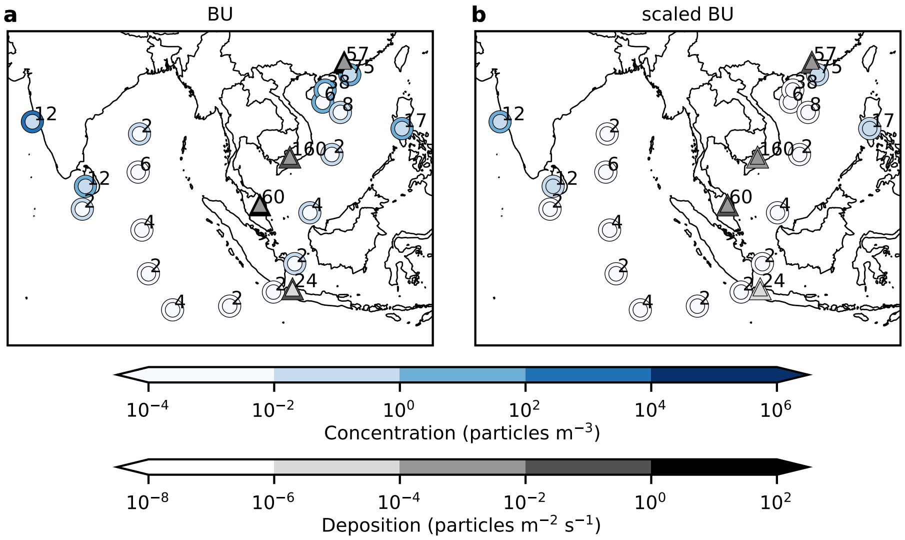

Supplementary Figure 14: Same as Supplementary Figure 6, but for the region of South Asia (SAS).

References

[35] Meijer, J. R., Huijbregts, M. A. J., Schotten, K. C. G. J. & Schipper, A. M. Global patterns of current and future road infrastructure. *Environmental Research Letters* **13**, 064006 (2018). URL https://iopscience.iop.org/article/10.1088/1748-9326/aabd42.

[36] Kole, P. J., Löhr, A. J., Van Belleghem, F. & Ragas, A. Wear and Tear of Tyres: A Stealthy Source of Microplastics in the Environment. *International Journal of Environmental Research and Public Health* **14**, 1265 (2017). URL https://www.mdpi.com/1660-4601/14/10/1265.

[37] Evangeliou, N. *et al.* Atmospheric transport is a major pathway of microplastics to remote regions. *Nature Communications* **11**, 3381 (2020). URL https://www.nature.com/articles/s41467-020-17201-9.

[38] Burghardt, T. E. *et al.* Microplastics and road markings: the role of glass beads and loss estimation. *Transportation Research Part D: Transport and Environment* **102**, 103123 (2022). URL https://doi.org/10.1016/j.trd.2021.103123.

[39] Burghardt, T. E. & Pashkevich, A. Road markings and microplastics – A critical literature review. *Transportation Research Part D: Transport and Environment* **119**, 103740 (2023). URL https://doi.org/10.1016/j.trd.2023.103740.

[40] Pyshyev, S., Gunka, V., Grytsenko, Y. & Bratychak, M. Polymer Modified Bitumen: Review. *Chemistry & Chemical Technology* **10**, 631–636 (2016). URL http://science2016.lp.edu.ua/chcht/polymer-modified-bitumen-review.

[41] Evangelou, I., Tatsii, D., Bucci, S. & Stohl, A. Atmospheric Resuspension of Microplastics from Bare Soil Regions. *Environmental Science & Technology* **58**, 9741–9749 (2024). URL https://pubs.acs.org/doi/10.1021/acs.est.4c01252.

[42] Bucci, S., Richon, C. & Bakels, L. Exploring the Transport Path of Oceanic Microplastics in the Atmosphere. *Environmental Science & Technology* **58**, 14338–14347 (2024). URL https://pubs.acs.org/doi/10.1021/acs.est.4c03216.

[43] Evangeliou, N., Tichý, O., Eckhardt, S., Zwaaftink, C. G. & Brahney, J. Sources and fate of atmospheric microplastics revealed from inverse and dispersion modelling: From global emissions to deposition. *Journal of Hazardous Materials* **432**, 128585 (2022). URL https://doi.org/10.1016/j.jhazmat.2022.128585.

[44] Kreider, M. L., Panko, J. M., McAtee, B. L., Sweet, L. I. & Finley, B. L. Physical and chemical characterization of tire-related particles: Comparison of particles generated using different methodologies. *Science of The Total Environment* **408**, 652–659 (2010). URL https://doi.org/10.1016/j.scitotenv.2009.10.016.

[45] Snilsberg, B., Myran, T., Uthus, N. & Aurstad, J. Evaluation of Different Laboratory Methods for Simulation of Pavement Wear and Road Dust Generation. *Road Materials and Pavement Design* **9**, 287–304 (2008). URL https://www.tandfonline.com/doi/full/10.1080/14680629.2008.9690170.

[46] Järlskog, I. *et al.* Concentrations of tire wear microplastics and other traffic-derived non-exhaust particles in the road environment. *Environment International* **170**, 107618 (2022). URL https://doi.org/10.1016/j.envint.2022.107618.

[47] Grigoratos, T. & Martini, G. Brake wear particle emissions: a review. *Environmental Science and Pollution Research* **22**, 2491–2504 (2015). URL http://link.springer.com/10.1007/s11356-014-3696-8.

[48] Brahney, J. *et al.* Constraining the atmospheric limb of the plastic cycle. *Proceedings of the National Academy of Sciences* **118**, 1–10 (2021). URL https://pnas.org/doi/full/10.1073/pnas.2020719118.

[49] Koelmans, A. A. *et al.* Risk assessment of microplastic particles. *Nature Reviews Materials* **7**, 138–152 (2022). URL https://www.nature.com/articles/s41578-021-00411-y.

[50] Chen, Q. *et al.* Aging simulation of thin-film plastics in different environments to examine the formation of microplastic. *Water Research* **202**, 117462 (2021). URL https://doi.org/10.1016/j.watres.2021.117462.

[51] Trainic, M. *et al.* Airborne microplastic particles detected in the remote marine atmosphere. *Communications Earth and Environment* **1**, 1–9 (2020). URL http://dx.doi.org/10.1038/s43247-020-00061-y.

[52] Liu, K. *et al.* Consistent Transport of Terrestrial Microplastics to the Ocean through Atmosphere. *Environmental Science & Technology* **53**, 10612–10619 (2019). URL https://pubs.acs.org/doi/10.1021/acs.est.9b03427.

[53] Allen, S. *et al.* Examination of the ocean as a source for atmospheric microplastics. *PLOS ONE* **15**, e0232746 (2020). URL https://dx.plos.org/10.1371/journal.pone.0232746.

[54] Liu, K. *et al.* Global inventory of atmospheric fibrous microplastics input into the ocean: An implication from the indoor origin. *Journal of Hazardous Materials* **400**, 123223 (2020). URL https://doi.org/10.1016/j.jhazmat.2020.123223.

[55] Ferrero, L. *et al.* Airborne and marine microplastics from an oceanographic survey at the Baltic Sea: An emerging role of air-sea interaction? *Science of The Total Environment* **824**, 153709 (2022). URL https://doi.org/10.1016/j.scitotenv.2022.153709.

[56] Ding, Y. *et al.* The abundance and characteristics of atmospheric microplastic deposition in the northwestern South China Sea in the fall. *Atmospheric Environment* **253**, 118389 (2021). URL https://doi.org/10.1016/j.atmosenv.2021.118389.

[57] Chen, Q. *et al.* Long-range atmospheric transport of microplastics across the southern hemisphere. *Nature Communications* **14**, 7898 (2023). URL https://www.nature.com/articles/s41467-023-43695-0.

[58] Li, C. *et al.* Enhanced impacts evaluation of Typhoon Sinlaku (2020) on atmospheric microplastics in South China Sea during the East Asian Summer Monsoon. *Science of The Total Environment* **806**, 150767 (2022). URL https://doi.org/10.1016/j.scitotenv.2021.150767.

[59] Wang, X. *et al.* Efficient transport of atmospheric microplastics onto the continent via the East Asian summer monsoon. *Journal of Hazardous Materials* **414**, 125477 (2021). URL https://doi.org/10.1016/j.jhazmat.2021.125477.

[60] Wang, X. *et al.* Atmospheric microplastic over the South China Sea and East Indian Ocean: abundance, distribution and source. *Journal of Hazardous Materials* **389**, 121846 (2020). URL https://doi.org/10.1016/j.jhazmat.2019.121846.

[61] Goßmann, I. *et al.* Occurrence and backtracking of microplastic mass loads including tire wear particles in northern Atlantic air. *Nature Communications* **14**, 3707 (2023). URL https://www.nature.com/articles/s41467-023-39340-5.

[62] Goßmann, I. *et al.* Unraveling the Marine Microplastic Cycle: The First Simultaneous Data Set for Air, Sea Surface Microlayer, and Underlying Water. *Environmental Science & Technology* **57**, 16541–16551 (2023). URL https://pubs.acs.org/doi/10.1021/acs.est.3c05002.

[63] Martynova, A. *et al.* Atmospheric microfibrous deposition over the Eastern Red Sea coast. *Science of The Total Environment* **907**, 167902 (2024). URL https://doi.org/10.1016/j.scitotenv.2023.167902.

[64] Allen, S. *et al.* Evidence of free tropospheric and long-range transport of microplastic at Pic du Midi Observatory. *Nature Communications* **12**, 7242 (2021). URL https://www.nature.com/articles/s41467-021-27454-7.

[65] Akhbarizadeh, R. *et al.* Suspended fine particulate matter (PM2.5), microplastics (MPs), and polycyclic aromatic hydrocarbons (PAHs) in air: Their possible relationships and health implications. *Environmental Research* **192**, 110339 (2021). URL https://doi.org/10.1016/j.envres.2020.110339.

[66] Abbasi, S. *et al.* Distribution and potential health impacts of microplastics and microrubbers in air and street dusts from Asaluyeh County, Iran. *Environmental*

Pollution **244**, 153–164 (2019). URL https://doi.org/10.1016/j.envpol.2018.10.039.

[67] Li, Y. *et al.* Airborne fiber particles: Types, size and concentration observed in Beijing. *Science of the Total Environment* **705**, 135967 (2020). URL https://doi.org/10.1016/j.scitotenv.2019.135967.

[68] González-Pleiter, M. *et al.* Occurrence and transport of microplastics sampled within and above the planetary boundary layer. *Science of the Total Environment* **761**, 143213 (2021). URL https://doi.org/10.1016/j.scitotenv.2020.143213.

[69] Gaston, E., Woo, M., Steele, C., Sukumaran, S. & Anderson, S. Microplastics Differ Between Indoor and Outdoor Air Masses: Insights from Multiple Microscopy Methodologies. *Applied Spectroscopy* **74**, 1079–1098 (2020). URL https://journals.sagepub.com/doi/10.1177/0003702820920652.

[70] Prata, J. C. *et al.* The importance of contamination control in airborne fibers and microplastic sampling: Experiences from indoor and outdoor air sampling in Aveiro, Portugal. *Marine Pollution Bulletin* **159**, 111522 (2020). URL https://doi.org/10.1016/j.marpolbul.2020.111522.

[71] Liu, K., Wang, X., Wei, N., Song, Z. & Li, D. Accurate quantification and transport estimation of suspended atmospheric microplastics in megacities: Implications for human health. *Environment International* **132**, 105127 (2019). URL https://doi.org/10.1016/j.envint.2019.105127.

[72] Abbasi, S. *et al.* Microplastics in the atmosphere of Ahvaz City, Iran. *Journal of Environmental Sciences (China)* **126**, 95–102 (2023). URL https://doi.org/10.1016/j.jes.2022.02.044.

[73] Zhu, X. *et al.* Airborne Microplastic Concentrations in Five Megacities of Northern and Southeast China. *Environmental Science & Technology* **55**, acs.est.1c03618 (2021). URL https://pubs.acs.org/doi/10.1021/acs.est.1c03618.

[74] Shruti, V. C., Kutralam-Muniasamy, G., Pérez-Guevara, F., Roy, P. D. & Martínez, I. E. Occurrence and characteristics of atmospheric microplastics in Mexico City. *Science of the Total Environment* **847**, 157601 (2022). URL https://doi.org/10.1016/j.scitotenv.2022.157601.

[75] Rao, W., Fan, Y., Li, H., Qian, X. & Liu, T. New insights into the long-term dynamics and deposition-suspension distribution of atmospheric microplastics in an urban area. *Journal of Hazardous Materials* **463**, 132860 (2024). URL https://doi.org/10.1016/j.jhazmat.2023.132860.

[76] Chandrakanthan, K., Fraser, M. P. & Herckes, P. Airborne microplastics in a suburban location in the desert southwest: Occurrence and identification challenges. *Atmospheric Environment* **298**, 119617 (2023). URL https://doi.org/10.1016/j.

atmosenv.2023.119617.

[77] Kyriakoudes, G. & Turner, A. Suspended and deposited microplastics in the coastal atmosphere of southwest England. *Chemosphere* **343**, 140258 (2023). URL https://doi.org/10.1016/j.chemosphere.2023.140258.

[78] Yuan, Z. *et al.* Vertical distribution and transport of microplastics in the urban atmosphere: New insights from field observations. *Science of the Total Environment* **895**, 165190 (2023). URL https://doi.org/10.1016/j.scitotenv.2023.165190.

[79] Xu, X. *et al.* Characterization of Microplastics in Clouds over Eastern China. *Environmental Science & Technology Letters* **11**, 16–22 (2024). URL https://pubs.acs.org/doi/10.1021/acs.estlett.3c00729.

[80] Romarate, R. A. *et al.* Breathing plastics in Metro Manila, Philippines: presence of suspended atmospheric microplastics in ambient air. *Environmental Science and Pollution Research* **30**, 53662–53673 (2023). URL https://doi.org/10.1007/s11356-023-26117-y.

[81] Sharaf Din, K., Khokhar, M. F., Butt, S. I., Qadir, A. & Younas, F. Exploration of microplastic concentration in indoor and outdoor air samples: Morphological, polymeric, and elemental analysis. *Science of the Total Environment* **908**, 168398 (2024). URL https://doi.org/10.1016/j.scitotenv.2023.168398.

[82] Liu, P. *et al.* Physicochemical characteristics of airborne microplastics of a typical coastal city in the Yangtze River Delta Region, China. *Journal of Environmental Sciences* **148**, 602–613 (2025). URL https://doi.org/10.1016/j.jes.2023.09.027.

[83] Morioka, T., Tanaka, S., Kohama-Inoue, A. & Watanabe, A. The quantification of the airborne plastic particles of 0.43–11 μm: Procedure development and application to atmospheric environment. *Chemosphere* **351**, 141131 (2024). URL https://doi.org/10.1016/j.chemosphere.2024.141131.

[84] Yuan, Z. *et al.* Atmospheric microplastics at a southern China metropolis: Occurrence, deposition flux, exposure risk and washout effect of rainfall. *Science of The Total Environment* **869**, 161839 (2023). URL https://doi.org/10.1016/j.scitotenv.2023.161839.

[85] Kaushik, A. *et al.* Identification and physico-chemical characterization of microplastics in marine aerosols over the northeast Arabian Sea. *Science of The Total Environment* **912**, 168705 (2024). URL https://doi.org/10.1016/j.scitotenv.2023.168705.

[86] Perera, K., Ziajahromi, S., Bengtson Nash, S., Manage, P. M. & Leusch, F. D. Airborne Microplastics in Indoor and Outdoor Environments of a Developing Country in South Asia: Abundance, Distribution, Morphology, and Possible

Sources. *Environmental Science & Technology* **56**, 16676–16685 (2022). URL https://pubs.acs.org/doi/10.1021/acs.est.2c05885.

[87] Liao, Z. *et al.* Airborne microplastics in indoor and outdoor environments of a coastal city in Eastern China. *Journal of Hazardous Materials* **417**, 126007 (2021). URL https://doi.org/10.1016/j.jhazmat.2021.126007.

[88] Guo, Z. *et al.* Characteristics, sources and potential ecological risk of atmospheric microplastics in Lhasa city. *Environmental Geochemistry and Health* **46**, 347 (2024). URL https://link.springer.com/10.1007/s10653-024-02125-w.

[89] Long, X. *et al.* Atmospheric Microplastics Emission Source Potentials and Deposition Patterns in Semi-Arid Croplands of Northern China. *Journal of Geophysical Research: Atmospheres* **129** (2024). URL https://agupubs.onlinelibrary.wiley.com/doi/10.1029/2024JD041546.

[90] Nafea, T. H., Shun Chan, F. K., Xu, Y., Xiao, H. & He, J. Unveiling the seasonal transport and exposure risks of atmospheric microplastics in the southern area of the Yangtze River Delta, China. *Environmental Pollution* **367**, 125567 (2025). URL https://doi.org/10.1016/j.envpol.2024.125567.

[91] Dris, R. *et al.* Microplastic contamination in an urban area: a case study in Greater Paris. *Environmental Chemistry* **12**, 592 (2015). URL http://www.publish.csiro.au/?paper=EN14167.

[92] Dris, R., Gasperi, J., Saad, M., Mirande, C. & Tassin, B. Synthetic fibers in atmospheric fallout: A source of microplastics in the environment? *Marine Pollution Bulletin* **104**, 290–293 (2016). URL https://doi.org/10.1016/j.marpolbul.2016.01.006.

[93] Cai, L. *et al.* Characteristic of microplastics in the atmospheric fallout from Dongguan city, China: preliminary research and first evidence. *Environmental Science and Pollution Research* **24**, 24928–24935 (2017). URL http://link.springer.com/10.1007/s11356-017-0116-x.

[94] Klein, M. & Fischer, E. K. Microplastic abundance in atmospheric deposition within the Metropolitan area of Hamburg, Germany. *Science of the Total Environment* **685**, 96–103 (2019). URL https://doi.org/10.1016/j.scitotenv.2019.05.405.

[95] Allen, S. *et al.* Atmospheric transport and deposition of microplastics in a remote mountain catchment. *Nature Geoscience* **12**, 339–344 (2019). URL https://www.nature.com/articles/s41561-019-0335-5.

[96] Roblin, B., Ryan, M., Vreugdenhil, A. & Aherne, J. Ambient Atmospheric Deposition of Anthropogenic Microfibers and Microplastics on the Western Periphery of Europe (Ireland). *Environmental Science & Technology* **54**, 11100–11108 (2020).

URL https://pubs.acs.org/doi/10.1021/acs.est.0c04000.

[97] Szewc, K., Graca, B. & Dołęga, A. Atmospheric deposition of microplastics in the coastal zone: Characteristics and relationship with meteorological factors. *Science of The Total Environment* **761**, 143272 (2021). URL https://doi.org/10.1016/j.scitotenv.2020.143272.

[98] Allen, S. *et al.* An early comparison of nano to microplastic mass in a remote catchment's atmospheric deposition. *Journal of Hazardous Materials Advances* **7**, 100104 (2022). URL https://doi.org/10.1016/j.hazadv.2022.100104.

[99] Brahney, J., Hallerud, M., Heim, E., Hahnenberger, M. & Sukumaran, S. Plastic rain in protected areas of the United States. *Science* **368**, 1257–1260 (2020). URL https://www.science.org/doi/10.1126/science.aaz5819.

[100] Wright, S. L., Ulke, J., Font, A., Chan, K. L. & Kelly, F. J. Atmospheric microplastic deposition in an urban environment and an evaluation of transport. *Environment International* **136**, 105411 (2020). URL https://doi.org/10.1016/j.envint.2019.105411.

[101] Kernchen, S. *et al.* Airborne microplastic concentrations and deposition across the Weser River catchment. *Science of The Total Environment* **818**, 151812 (2022). URL https://doi.org/10.1016/j.scitotenv.2021.151812.

[102] Hamilton, B. M. *et al.* Microplastics around an Arctic seabird colony: Particle community composition varies across environmental matrices. *Science of the Total Environment* **773**, 145536 (2021). URL https://doi.org/10.1016/j.scitotenv.2021.145536.

[103] Huang, Y. *et al.* Atmospheric transport and deposition of microplastics in a subtropical urban environment. *Journal of Hazardous Materials* **416**, 126168 (2021). URL https://doi.org/10.1016/j.jhazmat.2021.126168.

[104] Thinh, T. Q. *et al.* Preliminary assessment on the microplastic contamination in the atmospheric fallout in the Phuoc Hiep landfill, Cu Chi, Ho Chi Minh city. *Vietnam Journal of Science, Technology and Engineering* **62**, 83–89 (2020). URL https://vietnamscience.vjst.vn/index.php/VJSTE/article/view/352/248.

[105] Truong, T. N. S. *et al.* Microplastic in atmospheric fallouts of a developing Southeast Asian megacity under tropical climate. *Chemosphere* **272**, 129874 (2021). URL https://doi.org/10.1016/j.chemosphere.2021.129874.

[106] Purwiyanto, A. I. S. *et al.* The deposition of atmospheric microplastics in Jakarta-Indonesia: The coastal urban area. *Marine Pollution Bulletin* **174**, 113195 (2022). URL https://doi.org/10.1016/j.marpolbul.2021.113195.

[107] Abbasi, S. & Turner, A. Dry and wet deposition of microplastics in a semi-arid region (Shiraz, Iran). *Science of the Total Environment* **786**, 147358 (2021). URL https://doi.org/10.1016/j.scitotenv.2021.147358.

[108] Welsh, B., Aherne, J., Paterson, A. M., Yao, H. & McConnell, C. Atmospheric deposition of anthropogenic particles and microplastics in south-central Ontario, Canada. *Science of the Total Environment* **835**, 155426 (2022). URL https://doi.org/10.1016/j.scitotenv.2022.155426.

[109] Amato-Lourenço, L. F., dos Santos Galvão, L., Wiebeck, H., Carvalho-Oliveira, R. & Mauad, T. Atmospheric microplastic fallout in outdoor and indoor environments in São Paulo megacity. *Science of The Total Environment* **821**, 153450 (2022). URL https://doi.org/10.1016/j.scitotenv.2022.153450.

[110] Jenner, L. C. *et al.* Outdoor Atmospheric Microplastics within the Humber Region (United Kingdom): Quantification and Chemical Characterisation of Deposited Particles Present. *Atmosphere* **13**, 265 (2022). URL https://www.mdpi.com/2073-4433/13/2/265.

[111] Kernchen, S. *et al.* Atmospheric deposition studies of microplastics in Central Germany. *Air Quality, Atmosphere & Health* **17**, 2247–2261 (2024). URL https://link.springer.com/10.1007/s11869-024-01571-w.

[112] Liu, Z. *et al.* Distribution and possible sources of atmospheric microplastic deposition in a valley basin city (Lanzhou, China). *Ecotoxicology and Environmental Safety* **233**, 113353 (2022). URL https://doi.org/10.1016/j.ecoenv.2022.113353.

[113] Hee, Y. Y. *et al.* Atmospheric microplastic transport and deposition to urban and pristine tropical locations in Southeast Asia. *Science of the Total Environment* **902**, 166153 (2023). URL https://doi.org/10.1016/j.scitotenv.2023.166153.

[114] Abbasi, S. Microplastics washout from the atmosphere during a monsoon rain event. *Journal of Hazardous Materials Advances* **4**, 100035 (2021). URL https://doi.org/10.1016/j.hazadv.2021.100035.

[115] Jia, Q. *et al.* Atmospheric deposition of microplastics in the megalopolis (Shanghai) during rainy season: Characteristics, influence factors, and source. *Science of the Total Environment* **847**, 157609 (2022). URL https://doi.org/10.1016/j.scitotenv.2022.157609.

[116] Zhang, R. *et al.* Characteristics, sources and influencing factors of atmospheric deposition of microplastics in three different ecosystems of Beijing, China. *Science of The Total Environment* **883**, 163567 (2023). URL https://doi.org/10.1016/j.scitotenv.2023.163567.

[117] Edo, C. *et al.* A nationwide monitoring of atmospheric microplastic deposition. *Science of The Total Environment* **905**, 166923 (2023). URL https://doi.org/10.

1016/j.scitotenv.2023.166923.

[118] Lu, L. *et al.* Occurrence, influencing factors and sources of atmospheric microplastics in peri-urban farmland ecosystems of Beijing, China. *Science of The Total Environment* **912**, 168834 (2024). URL https://doi.org/10.1016/j.scitotenv.2023.168834.

[119] Parashar, N. & Hait, S. Plastic rain—Atmospheric microplastics deposition in urban and peri-urban areas of Patna City, Bihar, India: Distribution, characteristics, transport, and source analysis. *Journal of Hazardous Materials* **458**, 131883 (2023). URL https://doi.org/10.1016/j.jhazmat.2023.131883.

[120] Aves, A., Ruffell, H., Evangeliou, N., Gaw, S. & Revell, L. E. Modelled sources of airborne microplastics collected at a remote Southern Hemisphere site. *Atmospheric Environment* **325**, 120437 (2024). URL https://doi.org/10.1016/j.atmosenv.2024.120437.

[121] Beaurepaire, M., Gasperi, J., Tassin, B. & Dris, R. COVID lockdown significantly impacted microplastic bulk atmospheric deposition rates. *Environmental Pollution* **344**, 123354 (2024). URL https://doi.org/10.1016/j.envpol.2024.123354.

[122] Ankit, Y. *et al.* Atmospheric deposition of microplastics in an urban conglomerate near to the foothills of Indian Himalayas: Investigating the quantity, chemical character, possible sources and transport mechanisms. *Environmental Pollution* **361**, 124629 (2024). URL https://doi.org/10.1016/j.envpol.2024.124629.

[123] Chen, Y. *et al.* Atmospheric deposition of microplastics at a western China metropolis: Relationship with underlying surface types and human exposure. *Environmental Pollution* **355**, 124192 (2024). URL https://doi.org/10.1016/j.envpol.2024.124192.

[124] Elnahas, A. *et al.* Atmospheric Deposition of Microplastics in South Central Appalachia in the United States. *ACS ES&T Air* **2**, 64–72 (2025). URL https://pubs.acs.org/doi/10.1021/acsestair.4c00189.

[125] Illuminati, S. *et al.* Microplastics in bulk atmospheric deposition along the coastal region of Victoria Land, Antarctica. *Science of The Total Environment* **949**, 175221 (2024). URL https://doi.org/10.1016/j.scitotenv.2024.175221.

[126] Logvina, Y. *et al.* Microplastic Aerosol Contamination in Porto (Portugal). *Microplastics* **3**, 696–716 (2024). URL https://www.mdpi.com/2673-8929/3/4/43.
